# Synthesis of Platinum(II) Complexes with Some 1-Methylnitropyrazoles and In Vitro Research on Their Cytotoxic Activity

**DOI:** 10.3390/ph13120433

**Published:** 2020-11-28

**Authors:** Henryk Mastalarz, Agnieszka Mastalarz, Joanna Wietrzyk, Magdalena Milczarek, Andrzej Kochel, Andrzej Regiec

**Affiliations:** 1 Department of Organic Chemistry, Faculty of Pharmacy, Wrocław Medical University, 211A Borowska Street, 50-556 Wrocław, Poland; andrzej.regiec@umed.wroc.pl; 2 Faculty of Chemistry, The University of Wrocław, 14F Joliot-Curie Street, 50-383 Wrocław, Poland; a_mastalarz@interia.pl (A.M.); andrzej.kochel@chem.uni.wroc.pl (A.K.); 3 Hirszfeld Institute of Immunology and Experimental Therapy, Polish Academy of Sciences, 12 Rudolf Weigl Street, 53-114 Wrocław, Poland; joanna.wietrzyk@hirszfeld.pl (J.W.); magdalena.milczarek@hirszfeld.pl (M.M.)

**Keywords:** nitropyrazoles-Pt(II)complexes, synthesis, structural analysis, LogP, cellular platinum uptake, antiproliferative activity, normoxia-hypoxia, L-glutathione (GSH), cell cycle, X-ray crystallography

## Abstract

A series of eight novel platinum(II) complexes were synthesized by the reaction of the appropriate 1-methylnitropyrazole derivatives with K_2_PtCl_4_ and characterized by elemental analysis, ESI MS spectrometry, ^1^H NMR, ^195^Pt NMR, IR and far IR spectroscopy. Thermal isomerization of *cis*-dichloridobis(1-methyl-4-nitropyrazole)platinum(II) **1** to *trans*-dichloridobis(1-methyl-4-nitropyrazole)platinum(II) **2** has been presented, and the structure of the compound **2** has been confirmed by X-ray diffraction method. Cytotoxicity of the investigated compounds was examined in vitro on three human cancer cell lines (MCF-7 breast, ES-2 ovarian and A-549 lung adenocarcinomas) and their logP was measured using a shake-flask method. The *trans* complex **2** showed better antiproliferative activity than cisplatin for all the tested cancer cell lines. Additionally, *trans*-dichloridobis(1-methyl-5-nitropyrazole)platinum(II) **4** has featured a lower IC_50_ value than reference cisplatin against MCF-7 cell line. To gain additional information that may facilitate the explanation of the mode of action of tested compounds cellular platinum uptake, stability in L-glutathione solution, influence on cell cycle progression of HL-60 cells and ability to apoptosis induction were determined for compounds **1** and **2**.

## 1. Introduction

**Figure 1:**
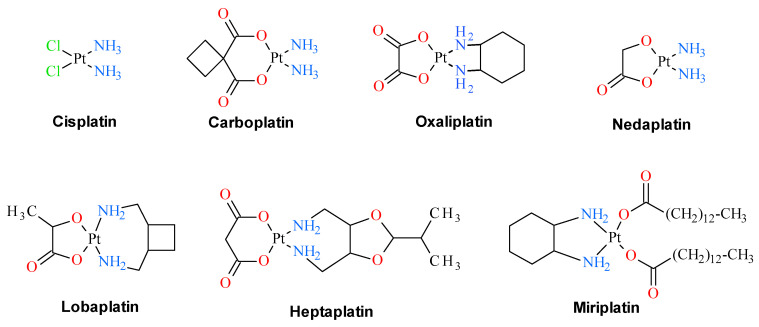
Clinically approved platinum drugs that are presently used in anticancer therapies.

Since the discovery of cisplatin cytostatic properties, platinum complexes have been widely used in modern medicine for the treatment of various solid tumors. Three of these compounds, namely cisplatin, oxaliplatin and carboplatin, are in clinical use as anti-cancer drugs. Four others, namely heptaplatin, nedaplatin, miriplatin and lobaplatin, have gained limited approval for their oncological purposes (Figure 1). In addition, a number of other platinum derivatives are currently under clinical trials. Despite being used medically for over 30 years, their detailed mechanism of antitumor action remains ambiguous, partly because these compounds display numerous intracellular targets. The anticancer activity of cisplatin is believed to arise from its interaction with DNA. Several cellular pathways are activated in response to this interaction, including recognition by repair enzymes, translation synthesis by polymerases, and induction of apoptosis. All these processes are suspected to be responsible for developing cisplatin resistance of tumor cells, which is the important factor limiting the effectiveness of cisplatin in medication in the treatment of cancer disease. Additionally, platinum based anticancer drugs are characterized by a narrow therapeutic index that results from their systemic toxicity which is tightly connected with their lack of tumor selectivity. To improve the efficacy of platinum-containing chemotherapeutic agents, there is still interest in the design and synthesis of this class of compounds with diminished systemic toxicity and maximized activity in tumors employing site-specific activation [1].

It is well known that tumor hypoxia provides a crucial difference between cancer cells and normal cells, offering a reductive environment for bio reducible prodrug activation. Tumor hypoxia has been shown in many studies to be responsible for treatment resistance and poor prognosis in various cancer types. Considering these facts, anaerobic reduction may be a promising route to achieve targeting and selectivity in anticancer drug design. In our investigation to obtain and test biologically novel compounds with potentially increased therapeutic effectiveness and diminished systemic side effects of cancer chemotherapy, a series of platinum(II) complexes with nitropyrazole ligands were designed as potential anticancer prodrugs containing a nitro group as a moiety susceptible to bioreductive activation. Several attempts to utilize the process of nitro group reduction as a trigger mechanism for the site-specific activation of potential anticancer prodrugs in hypoxic tumor tissues have been made with promising effects [2,3]. In 1984, the synthesis and biological activity of similar nitroheterocyclic complexes with Au(III), Pd(II), Rh(III), Pt(IV) and Pt(II) was patented and claimed to be cytotoxic, but no details of a structure (*cis* or *trans* geometry), biological activity, separation, purification, spectroscopic and physical properties of Pt(II) derivatives were given there [4].

**Figure 2:**
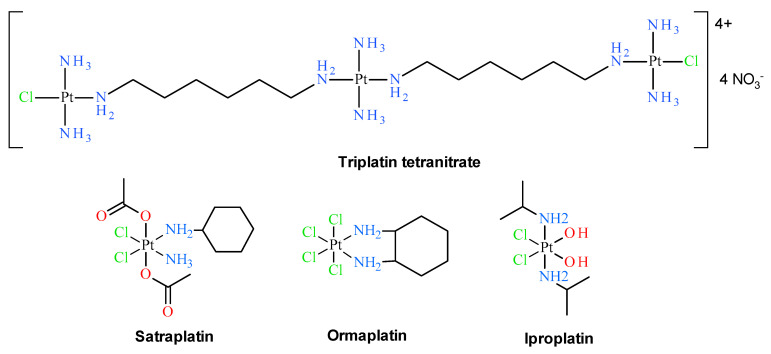
Polynuclear *trans*-platinum(II) and platinum(IV) complexes at various stages of clinical trials, not having official drug status.

Early classical structure-activity relationships for platinum coordination compounds established that only those with *cis* geometry possess the ability to inhibit cell growth. For example, cisplatin exhibits antitumor activity, whereas its *trans* isomer was proven to be inactive [5]. Additionally, the vast majority of cisplatin analogues with remarkable cytostatic properties contain at least one N–H group, which is claimed as a hydrogen-bond donor in the approach of the biological target, e.g., a DNA molecule [6]. Thus, typical platinum anticancer complexes have the general formula *cis*-[Pt(NHR_1_R_2_)_2_X_2_], in which R_1_ and R_2_ are organic moieties and X is a leaving group, usually a monovalent and biologically acceptable ion such as chlorido, nitrato or carboxylato ligands [7]. Although these findings can still be useful in the development of new platinum complexes with anticancer properties, starting in the early 1990s, numerous compounds have broken the rules mentioned above and have been synthesized and biologically tested with promising results [1,8,9,10,11,12]. Several classes of such non-classical (or non-conventional) platinum drugs can be distinguished, including *trans* platinum(II) analogs [11,12,13,14,15,16,17,18,19,20], Pt(IV) derivatives [21,22] polynuclear Pt complexes [23,24], Pt complexes with *N*-heterocyclic ligands [25], *N*-heterocyclic carbenes and cyclometallated Pt complexes [8], Pt(II) compounds with sulfur and phosphorus donors [26], compounds with leaving groups other than chlorido ligands [27]. However, it is necessary to emphasize that none of the *trans* Pt(II) and Pt(IV) complexes have been officially registered as anticancer drugs so far. The only promising polinuclear platinum(II) complex with *trans* moiety is triplatin (see Figure 2), which entered human clinical trials but further tests have been discontinued [24,28]. Satraplatin [21] (see Figure 2), which is an example of platinum(IV) complex, is still in advanced (phase III) clinical trials [28,29]. Clinical trials on other promising Pt(IV) complexes such as ormaplatin and iproplatin (see Figure 2) were also discontinued.

In 1990, Skov et al. [30] revealed the description of the synthesis and cytostatic properties of Pt(II) complexes of general formula *cis*-, *trans*-Pt(NH_3_)(L)Cl_2_, where L is a nitroazole ligand as a potential radiosensitizer [31]. A number of nitropyrazoles and known hypoxia activated drugs Etanidazole, Misonidazole or Metronidazole were used as ligands in these syntheses. These authors mentioned that *trans*-Pt(NH_3_Misonidazole)Cl_2_ is somewhat more active than its *cis* congener in cytotoxic tests, and both compounds were more active in hypoxia in comparison with aerobic conditions (however much less than cisplatin). However, from 1990, many *trans* complexes have been synthesized and have shown higher antitumor activity than their appropriate *cis* isomers; the results of biological tests which were published in this patent [30] intrigued us, and prompted us to synthesize and test a series of platinum(II) complexes with selected ligands from the broad group of various nitroazoles [32,33,34,35] coordinated as non-leaving ligands. Our expectations of possible cytotoxicity of such complexes were related to the previously claimed antiproliferative activity of similar compounds [4], despite the fact that they broke one important structure-activity relationship: they did not contain any N–H bonds in their amine ligands. According to our best knowledge, only very few Pt(II) complexes which show remarkable cytotoxicity and do not contain N–H bonds are known so far [11,36,37]. However, in these cases, the cytotoxic activity was usually due to the cytostatic nature of the ligands themselves or very high cellular accumulation level of such complexes, and there is no reason to expect any of the above effects from nitroazole derivatives. Thus, we considered the bioreduction of the nitro to the amine group to be the most probable preliminary hypothesis explaining the biological activity of Pt(II)-nitroazole complexes devoid of N–H bonds. The effect of nitro group bioreduction may be, however, not only the formation of N–H bonds. In its course, a number of chemically reactive intermediate products are generated, including free radicals and ion radicals capable of damaging nuclear DNA [38]. This is the reason for the mutagenic properties of nitro-containing antibacterial and antiprotozoal drugs, such as metronidazole, nitrofurans or nitazoxanide, and is the important factor in the mechanism of their biological action [39].

It is well known that both Pt(II) anticancer drugs and nitroazoles under hypoxic conditions have a proven ability to fix radiation-induced damage of cancer tissue. For this reason, our Pt(II) complexes containing nitroazole ligands [32,33,34,35] may also be considered as radiosensitizers [31,40].

The reduced reactivity towards endogenous thiols of sterically hindered Pt complexes containing a heterocyclic ligand has previously been reported [25], and therefore, we also expected the increased stability of our compounds under physiological conditions.

In the present paper, we have described some results of our research project concerning the Pt(II) complexes with various 1-methylnitropyrazoles only.

## 2. Results

### 2.1. Synthesis and Structural Analysis

**Figure 3:**
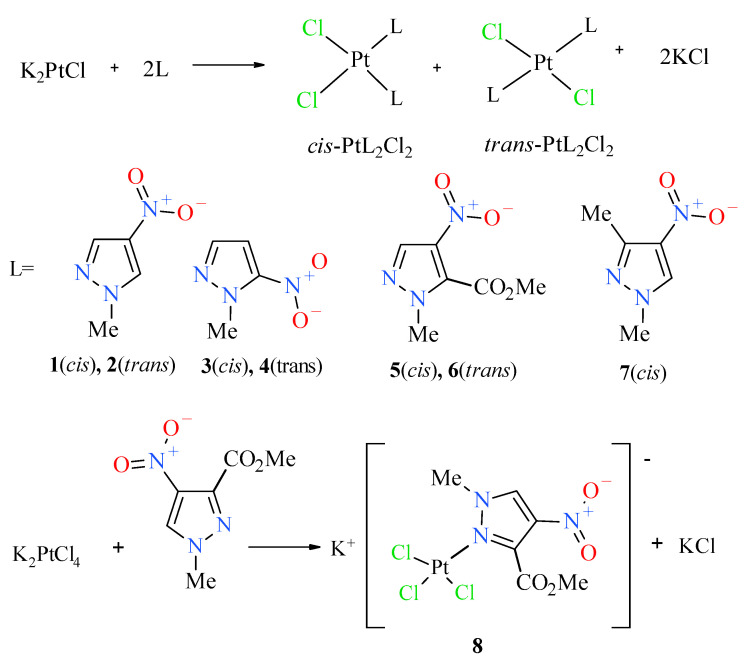
Scheme of synthesis of platinum(II) complexes **1**–**8**.

**Figure 4:**
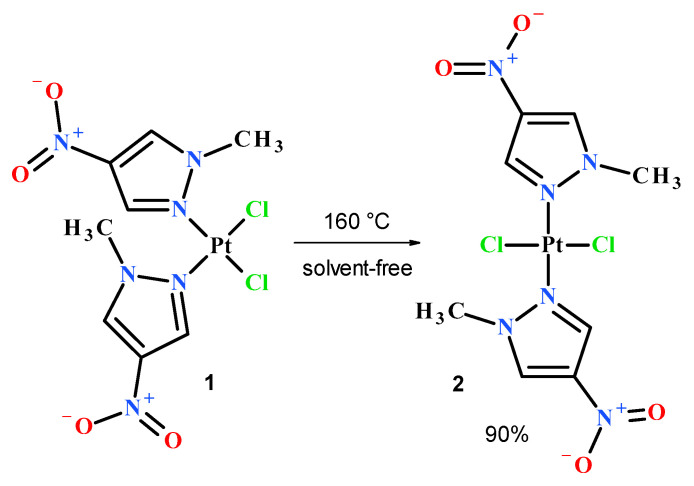
Thermal transformation of *cis* complex **1** into *trans* isomer **2**.

In order to obtain the desired nitropyrazole complexes (Figure 3), we synthesized six nitro 1-methylpyrazole derivatives, namely 1-methyl-3-nitropyrazole [32], 1-methyl-4-nitropyrazole [33], 1-methyl-5-nitropyrazole [32], 1,3-dimethyl-4-nitropyrazole [41], methyl 1-methyl-4-nitropyrazole-5-carboxylate [42] and methyl 1-methyl-4-nitropyrazole-3-carboxylate, according to the previously described procedures [42]. Using 1-methylated ligands allowed us to avoid the troublesome formation of additional isomers caused by the ring N–H tautomerism and should enhance the bioavailability of synthesized Pt complexes due to higher lipophilicity. Complexation reactions were carried out in the darkness at room temperature by mixing acetone or a water solution of two (2) molar equivalent of an appropriate ligand with a water solution of potassium tetrachloroplatinate (Figure 3). Reaction times were established by using TLC chromatography. Further workup of reaction mixtures depended on the ligand employed and the nature of formed products. Considering unsatisfactory yield (10%) of most active compound **2** during the standard procedure, we carried out an additional route of its synthesis through highly efficient (90%) thermal isomerization of its *cis* congener **1** [43] (Figure 4).

**Figure 5:**
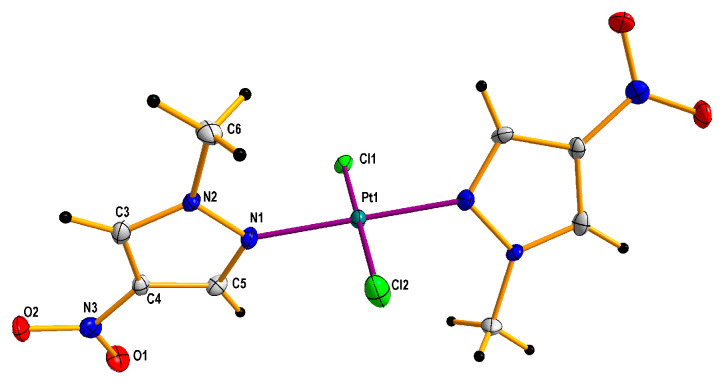
Molecular crystallographic structure determined on the basis of X-ray diffraction (XRD) for *trans*-dichloridobis(1-methyl-4-nitropyrazole) platinum(II) **2** di-acetone solvate. The solvent molecules were omitted for clarity. Thermal ellipsoids are drawn at the 50% probability level.

Additionally, it is worth adding that complexation with 1-methyl-3-nitropyrazole [32], was unsuccessful, probably because of the steric hindrance of both methyl and nitro groups that constrain access to free pair of electrons of pyrazole nitrogen. Besides, the electrostatic repelling of the approaching tetrachloroplatinate anion by electron dense oxygen atoms of the nitro group may also be an important factor that can hinder and/or prevent the formation of a complex. Besides, during the research it was also found that *cis* and *trans* complexes with 1-methyl-5-nitropyrazole easily isomerize in solutions, especially acetone ones, forming an equimolar mixture of isomers after some time. They are more stable in chloroform solutions, although in this case they are also isomerized, but much slower. All novel platinum(II) complexes were characterized by elemental analysis, ^1^H NMR spectroscopy (for the visualizations of the ^1^H NMR spectra, see Figures S22–S26 in Supplementary Material), IR and far IR spectroscopy (for the visualizations of the far IR spectra, see Figures S13–S20 in Supplementary Material) and ESI mass spectrometry (for the visualizations of the MS simulated and experimental spectra, see Figures S1–S12 in Supplementary Material), furthermore compound **2** was analyzed by X-ray diffraction (XRD) method (Figure 5) and ^195^Pt NMR spectroscopy (for the visualizations of the ^195^Pt NMR spectra, see Figure S21 in Supplementary Material). *cis* and *trans* congeners were distinguished by means of their far IR spectra at 400–300 cm^−1^ range because both *cis* and *trans* isomers exhibit strong Pt-Cl stretching vibration (at about ~350 cm^−1^), which is split into two bands only in the case of complexes with *cis* geometry. The reason is that both symmetric and antisymmetric valence vibrations of *cis* Pt(II)complexes are active, contrary to *trans* isomers in which case only antisymmetric vibration is active [44].

### 2.2. Lipophilicity

An important factor for the pharmacokinetics and the cellular accumulation of the investigated complexes, their lipophilicity was determined by measuring octanol/water partition coefficients (logP) with the shake-flask method [45]. The logP determination was carried out based on a previously described procedure [46], modified by us to obtain more accurate and repeatable results. The results are collected in Table 1. The value (−2.05) measured by us for cisplatin is within the range of previously reported values (−2.27 and −1.74) [45]. It is important to note that all tested *trans* isomers are characterized by higher logP values, and thus higher lipophilicity than their corresponding *cis* ones, which may be related to their zero electric dipole moments because of their centrosymmetric molecules. Besides, as shown in Table 1, all the compounds tested are more lipophilic than the reference cisplatin. The most noteworthy is the highest lipophilicity of *trans* complex **2** of all studied compounds.

### 2.3. Cellular Platinum Uptake

The uptake of novel Pt complexes **1**–**8** and reference cisplatin by MCF-7 breast cancer cells was measured after 4 h exposure at 10 µM concentration according to a previously described protocol [47] using Inductive Coupled Plasma Spectroscopy (ICP OES) for platinum determination. The results are presented in Table 1. Statistical analysis of data indicates that the compounds **3** and **4** have revealed a similar level of Pt accumulation in MCF-7 cells in comparison with cisplatin under the same conditions at the α = 0.05 threshold of significance level (*p*-value ≤ 0.05 level probability of statistical significance). However, the compounds (marked with stars in Table 1) **1**, **2**, **5**, **6** and **7** have been absorbed significantly (*p*-value ≤ 0.05 level probability of statistical significance) weaker than cisplatin by MCF-7 cells. It is necessary to strongly emphasize that the underlined values have a statistically significant lack of variance homogeneity (Fisher–Snedecor’s *F*-test, *F*_α/2_) with respect to reference drug variance, so Aspin–Welch’s *t*-test was applied in these cases. Compound **7** shows the statistically significant, weakest absorption of all tested compounds except the ionic derivative **8**. For ionic compound **8**, platinum uptake value was not determined due to Pt concentrations in the cell lysates being below the instrument detection limit (IDL = 10 μg/kg) specified by the manufacturer for the device used.

### 2.4. In Vitro Cytotoxic Activity

Determination of the cytotoxic activity of compounds tested was performed separately in the normoxia or hypoxia conditions to reveal their potentially bioreductive properties. The experiment was carried out on three human cancer cell lines: MCF-7 breast, ES-2 ovarian and A549 lung adenocarcinoma as well as normal murine embryonic fibroblast BALB/3T3 cell line. In order to facilitate the preparation of solutions of the tested compounds, their initial solutions for dilution were made by dissolving the compounds in acetone. For this purpose, it was not possible to use more polar aprotic solvents (DMSO, DMF) due to the rapid isomerization of some of the tested complexes under their influence. Moreover, as some research results have indicated that acetone is as good if not a better solvent for in vitro in cell growth studies than DMSO, due to its slightly lower toxicity [48].

The results are shown in Table 2. The most cytotoxic activity was revealed to be compound **2** with the lowest IC_50_ values in the case of all cancer cell lines compared to other tested compounds and referenced drug cisplatin. However, contrary to the rest of the tested compounds, this compound also showed the highest cytotoxic activity, similar to cisplatin, against normal BALB/3T3 cells. Most of the tested complexes appeared to be inactive or less active in hypoxia than in normoxia conditions. The exceptions were *trans* complex **2**, **4** and *cis* complex **3**. *trans* complex **2** did not show a difference in cytotoxic activity between normoxia and hypoxia conditions against all cell lines except MCF-7, where it appeared to be more active in normoxia. *Trans* complex **4** had similar cytotoxicity under both conditions only against ES-2 cell line, whereas in the case of rest cell lines it was more cytotoxic in normoxia. In contrast, *cis* complex **3** had similar cytotoxicity under both conditions only against MCF-7 cell line, but in the case of breast cancer (ES-2) it turned out more active in hypoxia as the only exception from the whole group of tested compounds. Additionally, it should be mentioned that compound **2** with higher antiproliferative activity than cisplatin and similar cytotoxicity against normal cells revealed a better selectivity index (SI; the ratio of cytotoxic concentration for healthy cells to cytotoxic concentration for cancer cells.) which was in the range 2.2–11.7 contrary to cisplatin (SI range 0.7–1). Due to the fact that *trans* complex **2** turned out to be the most active compound on all cancer cell lines tested, a pair of isomers, i.e., *cis* **1** and *trans* **2** were selected for further comparative research.

### 2.5. Reactivity with L-Glutathione (GSH)

**Figure 6:**
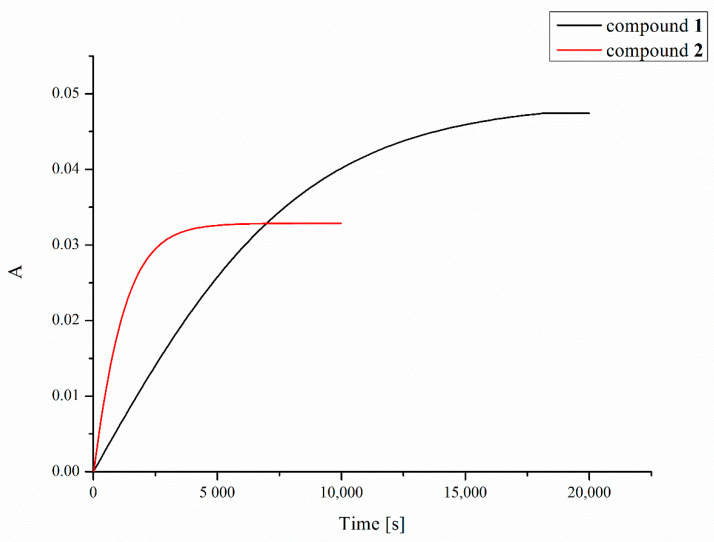
Time dependence of UV absorbance (at 260 nm) of compounds **1** (black line) and **2** (red line) in the presence of 2 mM of L-glutathione (GSH).

Platinum compounds have a strong ability to react with sulfur donor ligands. Hence, before a platinum-containing molecule reaches tumor cells, it may be deactivated in reactions with various endogenous sulfur-containing molecules. These side reactions are considered as playing an important role in mechanisms of tumor resistance to platinum drugs, their inactivation, and toxic side effects [49]. The two most important endogenous thiols to which platinum complexes can bind after intravenous administration or after they enter the cancer cell are reduced L-glutathione (GSH) and metallothionein (MT) [50]. In the present work, we investigated reactions of GSH with compounds **1** and **2**, measuring the UV absorbance at 260 nm (which indicates the formation of Pt–S and S–S bonds) as a function of time [51] using a double-beam spectrophotometer (Figure 6). Due to the poor solubility of the compounds in water, dioxane was used to prepare the stock solution of the compounds in order to more easily obtain a homogeneous solution, which was then diluted with an appropriate amount of water solution of L-glutathione (GSH). Despite the better solubility of tested complexes in acetone, dioxane was used in this experiment due to its lack of absorption of UV radiation at 260 nm in contrast to acetone that strongly absorbs at this wavelength preventing a correct measurement of absorbance changes. As shown in Figure 6, the increase in absorbance of the solution of complexes **1** and **2** and L-glutathione over time indicates clearly that a chemical reaction occurs resulting in a product that enhances the absorbance at the wavelength used.

The half-times of the reaction with 2 mM GSH, resulting mainly in the formation of Pt–S bonds, were found to be 83 and 13 min for compounds **1** and **2**, respectively. Increasing the concentration of L-glutathione to 4, 8 or 16 mM did not significantly affect the measurement results.

### 2.6. Cell Cycle and Cell Death Analysis

**Figure 7:**
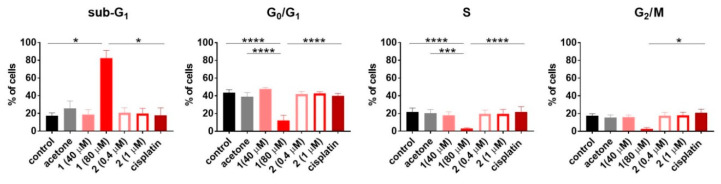
The cell cycle progression of HL-60 cells upon 48 h of exposure to compounds **1** and **2**. Cisplatin was applied as the reference compound at the concentration of 0.3 µM (0.1 µg/mL). Data represent the mean ± SD of at least three independent experiments. Statistical analysis was calculated using parametric one-way ANOVA followed by Dunnett’s (G_0_/G_1_ and S phase) and non-parametric Kruskal–Wallis (sub-G_1_ and G_2_/M phase) analysis, followed by Dunn’s multiple comparisons test (* *p* < 0.05, ** *p* < 0.01, *** *p* < 0.001, **** *p* < 0.0001).

**Figure 8:**
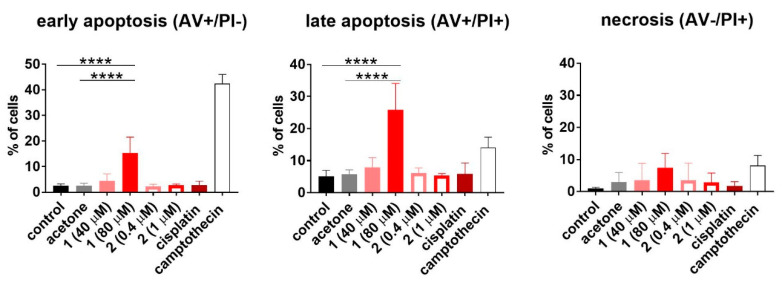
Detection of the apoptosis of HL-60 cells upon 48 h exposure to compounds **1** and **2**. Cisplatin was applied as the reference compound at the concentration of 0.3 µM (0.1 µg/mL). The cells were also exposed to camptothecin at the concentration of 5.7 µM (2 µg/mL) for 24 h as a positive control of apoptosis induction. The results are presented as the mean with SD calculated for at least three independent experiments. Statistical analysis was calculated using parametric one-way ANOVA followed by Dunnett’s (late apoptosis) and non-parametric Kruskal–Wallis analysis, followed by Dunn’s (early apoptosis) multiple comparisons test (**** *p* < 0.0001).

To establish how compounds **1** and **2** influence the cell cycle and cell death, the HL-60 human promyelocytic leukemia cell line was used. The results are outlined in the Figure 7 and Figure 8, respectively. Only compound **1** used at 80 µM concentration induced cell death (Sub-G_1_) of HL-60 cells. This led to disturbances in the cell cycle progression, expressed as a significant decrease in G_0_/G_1_ and S phase, compared to the untreated control (Figure 7). The double staining with Annexin V [52] and propidium iodide [53] confirmed the proapoptotic activity of compound **1** expressed as a statistically significant increase in early and late apoptotic cells (Figure 8).

## 3. Discussion

Synthesis of final Pt(II) complexes caused some problems only when 1-methyl-3 substituted pyrazoles were used as substrates in complexation reactions. One of the used ligands, namely 1-methyl-3-nitropyrazole, was unable to form a stable complex with the platinum(II) ion. In the case of 1,3-dimethyl-4-nitropyrazole, only *cis* isomer 7 formed with a very low yield. In sequence, methyl 1-methyl-4-nitropyrazole-3-carboxylate produced only ionic complex **8** with low yield. This was probably the result of steric hindrance caused by substituents placed tightly to the ring nitrogen donor atom.

The cytotoxic effects of the tested compounds **1**–**8** were determined in vitro against human A549 lung cancer, MCF-7 breast cancer, and ES-2 ovarian adenocarcinoma cell lines in normoxia and hypoxia conditions, according to established protocols. Out of an initial screening of the whole panel of eight substances, in vitro, four compounds **1**–**4**, particularly **2** and **4**, clearly showed greater and/or comparable activity to the reference drug (cisplatin) at least on some cell lines of cancer. However, only *trans* complex **2** (with 1-methyl-4-nitropyrazole ligands) showed higher cytotoxicity than cisplatin on all tumor lines tested. In contrast, *trans* complex **4** (with 1-methyl-5-nitropyrazole ligands) was more active than cisplatin only in the case of the MCF-7 line, in the tests for other cancer lines it proved to be weaker. In turn, *cis* complexes **1** and **3** have shown only comparable cytotoxicity to cisplatin on ES-2 and MCF-7 cell lines respectively; their cytotoxicity on the other lines was weaker. Remarkably, only the sole water-soluble ionic compound **8** turned out to be inactive in almost all cell lines except ES-2. The most sensitive cell line was MCF-7 breast cancer, in vitro growth of which was inhibited by compound **2** by a factor of about seven times and by compound **4** almost two times more strongly than by the reference cisplatin. The compounds **3**, **5** and **6** have shown statistically significant levels of activity against this tumor cell line, which was comparable to cisplatin. The most resistant tumor line appeared to be the human A549 lung cancer, on which only *trans* complex **2** (2.6 times larger) had a stronger effect than the reference cisplatin. The rest of the compounds were much weaker or inactive in this case. Against ES-2 ovarian adenocarcinoma cell lines, the *trans* **2** was the most active (more than cisplatin) and its *cis* isomer **1** had a statistically comparable activity to cisplatin. Other derivatives were much less active. It is worth noting that compound **2** outperformed the reference cisplatin showing 2.6 to about 12 times lower values of IC_50_. However, its cytotoxicity, measured on BALB/3T3 normal murine fibroblast cell line, was similar to the reference. Other tested compounds showed significantly much lower cytotoxicity (i.e., higher IC_50_) to normal healthy cells than cisplatin did. It is also worth noting that relatively small changes in the structure of non-leaving ligands, such as a change in the position of the nitro group in the non-leaving ligands, caused significant differences in the cytotoxicity of investigated compounds series.

All the compounds, including cisplatin, showed a greater or lesser dependence of cancer cell cytotoxicity on culturing conditions (normoxia versus hypoxia). This effect was most clearly manifested in the case of compounds **1** and **5–8** that unexpectedly exhibited a significantly lower cytotoxic activity in hypoxia conditions in the case of all tested cancer cell lines. In turn, the *trans* complex **4** with the 1-methyl-5-nitropyrazole moiety showed this symptom on all cell lines apart from ES-2. However, compound **2** and cisplatin exhibited this behavior for only one tumor line, i.e., MCF-7 and A549, for compound **2** and cisplatin, respectively. Exceptionally, only the *cis* complex **3** exhibited statistically relevant better activity in hypoxia, but only against the ES-2 cancer cell line. In the case of the A549 line, compound **3** showed lower activity in hypoxia, and in the case of line MCF-7 there was no statistical difference between the cytotoxic actions in both conditions. As shown above, most of the tested compounds exhibited diminished cytotoxic activity in hypoxic conditions in comparison with normoxia which would indicate their inability to be activated by bioreduction processes. In this way, the role of the nitro groups in antiproliferative properties of compound **2** became unclear.

Contrary to our expectations, there seems to be no simple correlation between cellular platinum uptake, lipophilicity and the biological activities of investigated compounds. The most active in vitro compound **2** has a moderate platinum uptake level, i.e., about twice to above three times lower than the reference cisplatin (see Table 1). On the other hand, complex **2** is the most lipophilic of all the tested compounds, which may suggest its exceptionally high affinity to cell membranes and strong interaction with them. In turn, complexes **3** and **4** containing 5-nitro-1-methylpyrazole ligands with much lower lipophilicity than most active compound **2** are the only ones with cellular uptake on the level of cisplatin. The unexpectedly low value of platinum uptake in the case of the most active compound **2** raised the suspicion that its interaction with DNA is not a key factor in its cytotoxic activity. In order to dispel these doubts and better understand the mechanism of action of the compound **2** and its *cis* isomer **1**, their influence on the cell cycle and cell death was examined. It turned out that both compounds did not show any substantial effect on the number of necrotic and late apoptic HL-60 human leukemia cells compared to 0.3 µM cisplatin and control, apart from **1** in excessive 80 µM concentration. Therefore, it should be assumed that the most likely cause of high cytotoxicity of compound **2**, despite its moderate level of cellular uptake and *trans* geometry, is direct or indirect interaction with DNA.

The measurement of the half-time of the reaction of the isomeric complexes **1** and **2** with a large molar excess of L-glutathione (83 and 13 min for compound **1** and **2**, respectively) revealed their diminished reactivity with endogenous thiols in comparison to the isomeric pair of cisplatin and transplatin (reaction half-times 66 and 4 min, respectively [54]. However, the *trans* complex **2** is inactivated by L-glutathione much faster than its *cis* congener **1** and cisplatin.

## 4. Conclusions

At this stage of the research, it is difficult to formulate unequivocal conclusions about the structure-activity relationship for the 1-methylnitropirazole Pt complexes. Although the mechanism of action of these compounds can be considered debatable, our preliminary pharmacological studies have ruled out two of the three common modes of cytotoxic action identified for metal complexes [55], namely the induction of apoptosis and inhibition of the cell cycle. Considering relatively poor “platination” of MCF-7 cells with our most active in vitro compound **2** and keeping in mind its *trans* geometry, it is also uncertain whether direct interaction with DNA could make a crucial contribution to its high cytotoxicity.

However, there are three structural factors whose influence on the biological properties of the investigated class of compounds seems unquestionable:

- The presence of an additional substituent in the 1-methylnitropyrazole ligand ring reduces in vitro cytotoxicity.
- The maximal possible distance of the nitro group from the coordination center of 1-methylnitropirazole ligand has a positive effect on the stability of its Pt complex.
- Generally, it can be observed that *trans* isomers are both more lipophilic and more active than their *cis* counterparts in the series of tested compounds.
- In most cases, the tested compounds were found to be inactive or less active under hypoxic conditions compared to normoxia.

## 5. Materials and Methods

### 5.1. General

The melting (m.p.) of the compounds were measured on Büchi M560 melting point apparatus (BÜCHI Labortechnik AG, CH-9230 Flawil/SG, Switzerland) and were uncorrected. Elemental analyses have been carried out by the Laboratory of Elemental Analyses, Faculty of Chemistry, Wroclaw University, with 2400 CHN elemental analyzer (Perkin-Elmer, Waltham, MA, USA). All Electrospray Mass Ionization (ESI) spectra were recorded with MicrOTOF-Q Mass Spectrometer (Bruker Daltonik GmbH, Bremen, Germany) using methanol (MeOH) as a solvent. The IR and far IR spectra (4000–40 cm^−1^) were measured with the Bruker 113v FTIR spectrophotometer (Bruker, Germany) in KBr pellets (650–4000 cm^−1^) or nujol mull (50–650 cm^−1^), respectively. ^1^H-NMR (300.15 MHz) spectra were recorded using ϕ5mm tubes and concentrations of about 20 mg of tested compounds in 0.6 mL of deuterated acetone-d_6_ and/or deuterated dimethylformamide (DMF-d_7_) with AMX Bruker NMR spectrometer (Bruker Analytische Messtechnik GmbH, Rheinstetten, Germany). Platinum concentrations were measured with iCAP 7400 Duo ICP-OES Analyzer (Thermo Fisher Scientific GmbH, Berlin, Germany) controlled with the integrated Thermo Scientific™ Qtegra™ Intelligent Scientific Data Solution™ (ISDS) software. The thin-layer chromatography method (TLC) was applied to monitor the reaction course as well as to confirm the purity of the synthesized compound. TLC-Al foils with fluorescent indicator 254 nm, silica gel matrix plates (Fluka Chemie GmbH, Buchs, Switzerland) for TLC were used, the eluting medium was chloroform-acetone (9:1 vol. ratio) or chloroform–methanol (95:5 vol. ratio), detection of the compounds on the chromatograms was done with 0.5% rubeanic acid (dithiooxamide) solution in acetone, UV light at 250 nm and/or by treatment with iodine vapors. Statistics were performed with Statistica (data analysis software system), version 13.3 (TIBCO Software Inc., Palo Alto, CA, USA (2017)), STATISTICA version 10 (StatSoft, Inc., Tulsa, OK, USA) and PTC Mathcad Express Prime 6.0.0.0. (Copyright 2019. PTC Inc.)

### 5.2. Synthesis of Platinum(II) Complexes

#### 5.2.1. Complexation with 1-Methyl-3-Nitropyrazole

A solution of 254 mg (2 mmol) of 1-methyl-3-nitropyrazole [32] in 11 mL of acetone was mixed with a solution of 415 mg (1 mmol) of K_2_PtCl_4_ in 10 mL of water and maintained in darkness at 25 °C. The reaction was monitored periodically by TLC and stopped after one month because of the remaining presence of unchanged starting material accompanied by a multi-component mixture of platinum-containing compounds. All attempts to isolate individual product, including column chromatography on SiO_2_, were unsuccessful.

#### 5.2.2. Complexation with 1-Methyl-4-Nitropyrazole

A solution of 254 mg (2 mmol) of 1-methyl-4-nitropyrazole [33,42] in 15 mL of warm water was mixed with a solution of 415 mg (1 mmol) of K_2_PtCl_4_ in 5 mL of water. The reaction mixture was maintained in darkness at 25 °C and monitored periodically by TLC until the absence of free ligand (app. 3 weeks). Then, the yellow precipitate was filtered off and dried, giving 480 mg (92%) of the crude mixture, which mainly consisted of two individual Pt complexes (TLC). These compounds were separated and purified using column chromatography on SiO_2_ with chloroform-acetone mixture as eluent giving 200 mg (yield 38%) of *cis-*dichloridobis(1-methyl-4-nitropyrazole)platinum(II) **1** (m.p. 180 °C (dec.)) and 50 mg (yield 10%) of *trans*-dichloridobis(1-methyl-4-nitropyrazole)platinum(II) **2** (less polar fraction, m.p. 180 °C (dec.)). Isomer **1** was soluble in acetone, slightly in chloroform, and poorly in water, diethyl ether, toluene and CCl_4_. Isomer **2** was very soluble in acetone, slightly in chloroform, diethyl ether, toluene and CCl_4_, but very poorly in water. Combustion and spectral analyses for the *cis* complex **1**: Elemental analysis for formula C_8_H_10_N_6_O_4_Cl_2_Pt of compound **1**: Calculated/Found (%): C 18.47/18.80, H 1.94/1.89, N 16.16/15.97, Cl 13.63/13.70. ^1^H NMR (300.15 MHz, acetone-d_6_): δ[ppm] 4.55 (s, 3H, N-CH_3_), 8.58 (s, 1H, H ar.), 8.98 (s, 1H, H ar.). ^195^Pt NMR (acetone-d_6_, external standard references K_2_PtCl_4_, δ[ppm] = −1618): δ[ppm] = −2091. IR, far IR ν[cm^−1^]: 1520(ν_as_ NO_2_), 1321(ν_s_ NO_2_), 817(def. NO_2_), 450(ν_as_N-Pt) 349(ν_s_Cl-Pt), 341(ν_as_Cl-Pt). Combustion and spectral analyses for the *trans* complex **2:** Elemental analysis for formula C_8_H_10_N_6_O_4_Cl_2_Pt of the *trans*-complex **2**: Calculated/Found (%): C 18.47/18.76, H 1.94/1.84, N 16.16/15.86, Cl 13.63/13.93. ^1^H NMR (300.15 MHz, acetone-d_6_): δ[ppm] 5.00(s, 3H, N-CH_3_), 9.04(s, 1H, H ar.), 9.44(s, 1H, H ar.). ^195^Pt NMR (acetone-d_6_, external standard reference K_2_PtCl_4_, δ[ppm] = −1618): δ[ppm] = −2119. IR, far IR ν[cm^−1^]: 1521(ν_as_ NO_2_), 1325(ν_s_ NO_2_), 821(def. NO_2_), 451(ν_as_N-Pt), 350(ν_as_Cl-Pt). Analysis of Mass Spectrum (ESI-MS) of both complexes **1** and **2**: The calculated value of the parent peak mass for the formula C_8_H_10_N_6_O_4_Cl_2_Pt was 518.9789 u. ESI MS (positive ionization) of **1** and **2** revealed, amongst others, following peaks given as a ratio *m/z* [u/e]: 541.9586 [PtL_2_Cl_2_+Na]^+^-quasi-molecular ion peak, 557.9331 [PtL_2_Cl_2_+K]^+^-quasi-molecular ion peak, where L = 1-methyl-4-nitropyrazole. ESI MS (negative ionization) of **1** and **2** revealed, amongst others, the following peak given as a ratio *m/z* [u/e]: 517.9561 [PtL_2_Cl_2_-H]^−^ where L = 1-methyl-4-nitropyrazole.

#### 5.2.3. Thermal Isomerization of *cis*-Complex **1** into its *trans* Isomer **2** Conducted in Solid State

For this process, 300 mg of pulverized compound **1** was placed in a round bottom flask and vigorously stirred at 160 °C for 2 h. The resulting pale grey solid was extracted several times with hot acetone, giving 270 mg (yield 90%) of pure (TLC) compound **2** after solvent evaporation.

#### 5.2.4. Complexation with 1-Methyl-5-Nitropyrazole

A solution of 254 mg (2 mmol) of 1-methyl-5-nitropyrazole [32] in 3 mL of acetone was mixed with a solution of 415 mg (1 mmol) of K_2_PtCl_4_ in 5 mL of water and maintained in darkness at 25 °C until the reaction completion (TLC, app. 3 days). Then, the yellow precipitate (170 mg, yield 32%) consisting of two individual Pt complexes *cis*-dichloridobis(1-methyl-5-nitropyrazole)platinum(II) (**3**) and *trans*-dichloridobis(1-methyl-5-nitropyrazole)platinum(II) (**4**) was filtered off and dried. Separation of these compounds by column chromatography on SiO_2_ with chloroform–methanol 8:1 solution did not lead to spectral pure isomers due to gradual isomerization of individual compounds in their solutions, resulting in binary starting composition (app. 1:1 molar) after several hours. Isomerization of the complexes **3** and **4** occurred slowly in chloroform or acetone solutions but much faster in more polar aprotic solvents (DMSO, DMF). Combustion and spectral analyses for the *cis* complex **3:** Elemental analysis for formula C_8_H_10_N_6_O_4_Cl_2_Pt of compound **3**: Calculated/Found (%): C 18.47/17.97, H 1.94/1.66, N 16.16/16.50, Cl 13.63/13.87. ^1^H NMR (300.15 MHz, acetone-d_6_): δ[ppm] 4.83(s, 3H, N-CH_3_), 7.34(s, 1H, H ar.), 8.10(s, 1H, H ar.). IR, far IR ν[cm^−1^]: 1560(ν_as_NO_2_), 1364(ν_s_NO_2_), 829(def. NO_2_), 349(s, ν_s_Cl-Pt), 344(s, ν_as_Cl-Pt). Combustion and spectral analyses for the *trans* complex **4:** Elemental analysis for formula C_8_H_10_N_6_O_4_Cl_2_Pt of *trans*-complex **4**: Calculated/Found (%): C 18.47/18.14, H 1.94/1.65, N 16.16/15.89, Cl 13.63/13.82. ^1^H NMR (300.15 MHz, acetone-d_6_): δ[ppm] 4.84(s, 3H, N-CH_3_), 7.32(s, 1H, H ar.), 8.26(s, 1H, H ar.). IR, far IR ν[cm^−1^]: 1560 (ν_as_NO_2_), 1362(ν_s_NO_2_), 832(def. NO_2_), 332 (s, ν_as_Pt-Cl). Analysis of Mass Spectrum (ESI-MS) of both complexes **3** and **4**: The calculated value of the parent peak mass for the formula C_8_H_10_N_6_O_4_Cl_2_Pt was 518.9789 u. ESI MS (positive ionization) of **3** and **4** revealed, amongst others, the following peaks given as a ratio *m/z* [u/e]: 541.9575 [PtL_2_Cl_2_+Na]^+^-quasi-molecular ion peak, 557.9326 [PtL_2_Cl_2_+K]^+^-quasi-molecular ion peak, where L = 1-methyl-5-nitropyrazole. ESI MS (negative ionization) of **3** and **4** revealed, amongst others, the following peak given as a ratio *m/z* [u/e]: 517.9555 [PtL_2_Cl_2_-H]^-^-quasi-molecular ion peak, where L = 1-methyl-5-nitropyrazole.

#### 5.2.5. Complexation with Methyl 1-Methyl-4-Nitropyrazole-5-Carboxylate

A solution of 370 mg (2 mmol) of methyl 1-methyl-4-nitropyrazole-5-carboxylate [42] in 7 mL of acetone was mixed with a solution of 415 mg (1 mmol) of K_2_PtCl_4_ in 10 mL of water and maintained in darkness at 25 °C for 1 month. The resulting yellow precipitate was filtered off and washed with chloroform to remove unchanged starting material, giving 500 mg of crude product which mainly consisted of two individual Pt compounds (TLC).

These compounds were separated and purified using column chromatography on SiO_2_ with chloroform-acetone mixture as eluent, giving 170 mg (27%) of *cis-*dichloridobis(methyl 1-methyl-4-nitropyrazole-5-carboxylate)platinum(II) (**5**, less polar fraction) and 50 mg (8%) of *trans*-dichloridobis(methyl 1-methyl-4-nitropyrazole-5-carboxylate)platinum(II) (**6**). Combustion and spectral analyses for the *cis* complex **5:** Elemental analysis for formula C_12_H_14_N_6_O_8_Cl_2_Pt of compound **5**: Calculated/Found (%): C 22.65/22.42, H 2.22/2.31, N 13.21/12.98, Cl 11.14/10.85. ^1^H NMR (300.15 MHz, acetone-d_6_): δ[ppm] 4.05(s, 3H, O-CH_3_), 4.54(s, 3H, N-CH_3_), 8.46(s, 1H, H ar.). IR, far IR ν[cm^−1^]: 1742(s, νC=O ester), 1533(ν_as_NO_2_), 1278(ν_s_NO_2_), 847(def. NO_2_), 350(s, ν_s_Cl-Pt), 336 (s, ν_as_Cl-Pt). Combustion and spectral analyses for the *trans* complex **6:** Elemental analysis for formula C_12_H_14_N_6_O_8_Cl_2_Pt of *trans*-complex **6**: Calculated/Found (%): C 22.65/22.36, H 2.22/2.48, N 13.21/12.90, Cl 11.14/11.34. ^1^H NMR (300.15 MHz, acetone-d_6_): δ[ppm] 4.05(s, 3H, O-CH_3_), 4.65(s, 3H, N-CH_3_), 9.06(s, 1H, H ar.). IR, far IR ν[cm^−1^]: 1745(s, νC=O ester), 1531(ν_as_NO_2_), 1277(ν_s_NO_2_), 846(defNO_2_), 348(br, ν_as_Pt-Cl). Analysis of Mass Spectrum (ESI-MS) of both complexes **5** and **6**: The calculated value of the parent peak mass for the formula C_12_H_14_N_6_O_8_Cl_2_Pt was 634.9898 u. ESI MS (positive ionization) of **5** and **6** revealed, amongst others, the following peaks given as a ratio *m/z* [u/e]: 657.9667 [PtL_2_Cl_2_+Na]^+^-quasi-molecular ion peak, 673.9388 [PtL_2_Cl_2_+K]^+^-quasi-molecular ion peak, where L = methyl 1-methyl-4-nitro-5-pyrazolecarboxylate. ESI MS (negative ionization) of **5** and **6** revealed, amongst others, the following peak given as a ratio *m/z* [u/e]: 633.9695 [PtL_2_Cl_2_-H]^−^-quasi-molecular ion peak, where L = methyl 1-methyl-4-nitro-5-pyrazolecarboxylate.

#### 5.2.6. Complexation with 1,3-Dimethyl-4-Nitropyrazole

A solution of 282 mg (2 mmol) of 1,3-dimethyl-5-nitropyrazole [41] in 10 mL of warm water was mixed with a solution of 415 mg (1 mmol) of K_2_PtCl_4_ in 5 mL of water and maintained in darkness at 25 °C until the reaction completion (TLC, app. 2 weeks), but only a small amount of precipitate occurred. The reaction mixture was evaporated to dryness and the solid residue was extracted several times with acetone, giving 450 mg of multi component yellow crystal mass. After purification using column chromatography on SiO_2_ with chloroform–acetone mixture as eluent, only a small amount (ca 50 mg, 9%) of *cis*-dichloridobis(1,3-dimethyl-4-nitropyrazole)platinum(II) (**7**) was obtained. Combustion and spectral analyses for the *cis* complex **7:** Elemental analysis for formula C_10_H_14_N_6_O_4_Cl_2_Pt of the compound **7**: Calculated/Found (%): C 21.83/21.59, H 2.93/3.21, N 15.27/15.20, Cl 12.89/13.13. ^1^H NMR (300.15 MHz, dimethylformamide - DMF-d_7_): δ[ppm] 3.06 (s, 3H, Ar-CH_3_), 4.45- 4.48 (s, 3H, N-CH_3_), 9.19 (s, 1H, H ar.). IR, far IR ν[cm^−1^]: 1547 (ν_as_NO_2_), 1346(vs, NO_2_), 847(s, def. NO_2_), 348(s, ν_s_Cl-Pt), 344 (s, ν_as_Pt-Cl). Analysis of Mass Spectrum (ESI-MS): The calculated value of the parent peak mass for the formula C_10_H_14_N_6_O_4_Cl_2_Pt was 547.0102 u. ESI MS (positive ionization) revealed, amongst others, the following peaks given as a ratio *m/z* [u/e]: 570.9837 [PtL_2_Cl_2_+Na]^+^-quasi-molecular ion peak, 585.9690 [PtL_2_Cl_2_+K]^+^-quasi-molecular ion peak, where L = 1,3-dimethyl-4-nitropirazole.

#### 5.2.7. Complexation with Methyl 1-Methyl-4-Nitropyrazole-3-Carboxylate

A solution of 170 mg (0.92 mmol) of methyl 1-methyl-4-nitropyrazole-3-carboxylate [42] in 6 mL of acetone was mixed with a solution of 190 mg (0.46 mmol) of K_2_PtCl_4_ in 10 mL of water and maintained in darkness at 25 °C for 1 month. The resulting precipitate consisting mostly of unchanged starting material was filtered off and the filtrate was evaporated to dryness and extracted with acetone giving, after evaporation, 190 mg of solid residue. Further purification by column chromatography on SiO_2_ with chloroform–acetone 3:1 as eluent gave 80 mg (15%) of potassium trichlorido(3-carboxymethyl-1-methyl-4-nitropirazole)platinate(II) **8**. Elemental analysis for formula KC_6_H_7_N_3_O_4_Cl_3_Pt of compound **8**: Calculated/Found (%): C 13.71/13.58, H 1.34/1.50, N 7.99/7.69, Cl 20.23/19.95. ^1^H NMR (300.15 MHz, acetone-d_6_): δ[ppm] 4.03(s, 3H, O-CH_3_), 4.57(s, 3H, N-CH_3_), 8.96(s, 1H, H ar.). IR, far IR ν[cm^−1^]: 1743 (vs, νC=O ester), 1550 (ν_as_NO_2_), 1261 (s, ν_s_NO_2_), 857(def. NO_2_), 332 (b, νPt-Cl). Analysis of Mass Spectrum (ESI-MS): The calculated value of the parent peak mass for the formula KC_6_H_7_N_3_O_4_Cl_3_Pt was 523.8782 u. ESI MS (negative ionization) revealed, amongst others, the following peaks given as a ratio *m/z* [u/e]: 484.9156 [PtLCl_3_]^−^-fragmentation ion peak (trichlorido(3-carboxymethyl-1-methyl-4-nitropirazole)platinate(II) anion), 448.9368 [PtLCl_2_-H]^−^-fragmentation ion peak, where L = methyl 1-methyl-4-nitro-3-pyrazolecarboxylate.

### 5.3. Single Crystal X-ray Structure Determination of **2**

The crystal of compound **2** suitable for single-crystal X-ray diffraction analysis was obtained by solvent evaporation of its acetone solution. Crystallographic measurements were collected with Κ-geometry diffractometers: Xcalibur Gemini with graphite monochromatized Mo-Kα radiation (λ = 0.71073 Å) at 100(2) K, using an Oxford Cryosystems cooler. Data collection, cell refinement, data reduction and analysis were carried out with CrysAlisPro [56]. Analytical absorption correction was applied to data with the use of CrysAlisPro. The crystal structures were solved using SHELXT [57] and refined on F2 by a full-matrix least squares technique with SHELXL-2016 [57] with anisotropic thermal parameters for all the ordered non-H atoms. In the final refinement cycles, H atoms were repositioned in their calculated positions and treated as riding atoms, with C–H = 0.95–0.98 Å, and with Uiso (H) = 1.2Ueq (C) for CH and 1.5Ueq(C) for CH_3_. Crystal data and details of data collection and refinement procedures were collected and are presented in Table 3. Figure 5 was made using DIAMOND program [58].

### 5.4. Determination of logP by Shake-Flask Method

Weighted amounts of the tested platinum complex were suspended by 10 min sonification in 0.9% NaCl water solution, which was previously saturated with n-octanol, and mixed by shaking for 1 h. A simultaneously similar procedure was performed using 5 mL of n-octanol pre-saturated with 0.9% NaCl. Afterward, both suspensions were mixed together, shaken for an additional 1 h, and centrifuged for 5 min. After phase separation and evaporation in vacuo of 1 mL of sample to dryness, the final concentration of Pt was determined by ICP-OES (λ = 265.945 nm) and the partition coefficients were calculated. For the analysis, all samples were dissolved 1:1000 in 2.5% HCl. Each measurement was repeated four times and the final logP was calculated as the arithmetic mean of the four values. Directly after the experiment, all organic and water phases were tested (TLC) for their homogeneity, but no symptoms of decomposition or isomerization of Pt compounds were detected [45,46].

### 5.5. Reaction with L-Glutathione

The solutions of compounds **1** or **2** in dioxane (30 µL) at 60 mM concentration were added to 3 mL of 2 mM solution of GSH (reduced L-glutathione) previously saturated with argon in the medium containing 10 mmol/L NaCl and 10 mmol/L Tris xHCl buffer, pH 7.4, warmed to 37 °C, reaching a final concentration of 6 µM of the platinum complex. The reaction progress during incubation at 37 °C was monitored using UV absorption spectrometry at 260 nm. In this experiment, dioxane was used to dissolve tested Pt complexes due to its lack of UV absorption at the wavelength used. Saturation of GSH with argon was carried out to remove oxygen from the solution and thus prevent the formation of S–S bonds that could distort the measurement results.

### 5.6. Cell Culture

Three human cancer cell lines: MCF-7 (breast adenocarcinoma), ES-2 (ovarian adenocarcinoma), A549 (lung adenocarcinoma) as well as also one normal cell line BALB/3T3 (murine embryonic fibroblast) were used to evaluate the cytotoxic activity of platinum derivatives. The HL-60 cell line (human acute promyelocytic leukemia) was used to evaluate the cell cycle distribution and death of cells after exposure to selected compounds. The 4T1 cell line (mouse mammary gland cancer) was used to evaluate the therapeutic effect of selected platinum derivatives.

MCF-7, ES-2, A549, BALB/3T3, and 4T1 cell lines were purchased from the American Type Culture Collection (ATCC Rockville, MD, USA), HL-60 cell line—from European Type Culture Collection by courtesy of Professor Spik and Dr. Mazurier (Laboratory of Biological Chemistry USTL, Lille, France). All the cell lines were maintained at the Hirszfeld Institute of Immunology and Experimental Therapy, Polish Academy of Sciences (HIIET, PAS), Wroclaw, Poland.

The MCF-7 cells were cultured in Eagle medium supplemented with 2 mM L-glutamine, 1.5 g/L bicarbonate, 1 mM sodium pyruvate, amino acids and insulin (all from Sigma-Aldrich, Steinheim, Germany) and 10% (*v/v*) fetal bovine serum (FBS) (Thermo Fisher Scientific, Finland). The ES-2 cells were cultured in RPMI 1640 medium supplemented with 4 mM L-glutamine (Sigma-Aldrich, Steinheim, Germany) and 10% (*v/v*) (FBS) (Thermo Fisher Scientific, Finland). The A-549 cells were cultured in 1:1 (*v/v*) mixture of RPMI-1640 and Opti-MEM medium (both from HIIET Wroclaw, Poland) supplemented with 2 mM L-glutamine (Sigma-Aldrich, Steinheim, Germany) and 5% (*v/v*) (FBS) (Thermo Fisher Scientific, Finland).

The BALB/3T3 cells were cultured in Dulbecco medium (Gibco, Darmstadt, Germany), supplemented with 10% FBS (*v/v*) (Thermo Fisher Scientific, Finland), 4.5 g/L glucose, and 4 mM L-glutamine (Sigma-Aldrich, Steinheim, Germany). The HL-60 cells were cultured in ISCOVE medium (HIIET, Wroclaw, Poland) containing 10% FBS (*v/v*) (Thermo Fisher Scientific, Vantaa, Finland) and 2 mM L-glutamine (Sigma-Aldrich, Steinheim, Germany). The 4T1 cells were cultured in 1:1 (*v/v*) mixture of RPMI-1640 and Opti-MEM medium (both from HIIET Wroclaw, Poland), supplemented with 5% FBS (*v/v*) (Thermo Fisher Scientific, Finland), 4.5 g/L glucose, 2 mM glutamine, and 1.0 mM sodium pyruvate (all from Sigma-Aldrich, Steinheim, Germany). All culture media contained antibiotics: 100 U/mL penicillin (Sigma-Aldrich, Steinheim, Germany) and 100 μg/mL streptomycin (Polfa-Tarchomin, Warsaw, Poland).

All the cell lines were cultured at 37 °C in a humid atmosphere containing 5% CO_2_. Additionally, in the case of cytotoxicity assay with hypoxic conditions, the cells (MCF-7, ES-2, A549) were maintained at 37 °C in a humid atmosphere containing 5% CO_2_ and 1% O_2_ during the entire experiment.

### 5.7. Compounds Preparation to In Vitro Studies

In the case of in vitro studies, the solutions of all platinum derivatives studied were prepared ex tempore for each test by dissolving them in acetone and then suspending them in the culture medium (1:9 (*v/v*), respectively) to reach the stock 1 mg/mL solution.

### 5.8. In Vitro Cytotoxicity Assay

The stock solutions were diluted in a culture medium to reach the final concentrations of 100 to 0.1 μg/mL; only the most active compound **2** was tested in 1 to 0.001 μg/mL concentrations. Twenty-four hours before adding the tested compounds, all cell lines were seeded in 96-well plates (SARSTEDT AG & Co, Nümbrecht Germany) in an appropriate media with 10^4^ cells per well. All cell lines were exposed to each compound tested at four different concentrations for 72 h. Cells were also exposed to the reference drug cisplatin (EBEWE Pharma GmbH Nfg. KG, Unterach am Attersee, Austria) and 1-methyl-4-nitropyrazole [31] ligand to ensure its inactivity. Additionally, all cell lines were exposed to acetone (Avantor Performance Materials, Gliwice, Poland) (the solvent used for tested compounds) at concentrations corresponding to those present in tested compounds’ dilutions.

### 5.9. Sulforhodamine B Assay

After 72 h of cell incubation with the compounds tested, the sulforhodamine B (SRB) assay was performed as described by Shekan et al. [59] with minor modifications. The cells were fixed in situ by adding 50 µL per well of cold 50% trichloroacetic acid (Avantor Performance Materials, Gliwice, Poland) and were incubated at 4 °C for one hour. Following that, wells were washed five times with water and air dried. Next, 50 µL of 0.4% solution of sulforhodamine B (Sigma-Aldrich, Steinheim, Germany) in 1% acetic acid (Avantor Performance Materials, Gliwice, Poland) were added to each well and plates were incubated at room temperature for 0.5 h. After incubation time, the unbound dye was removed by washing plates five times with 1% acetic acid, whereas stains bound to cells was solubilized with 10 mM Tris base (Sigma-Aldrich, Steinheim, Germany). The absorbance of each solution was read by the Multiskan RC photometer (Labsystems Diagnostics Oy, Vantaa, Finland) at a 540 nm wavelength. Results are presented as mean IC_50_ (concentration of the compound tested that inhibits cell proliferation by 50%) ± standard deviation. IC_50_ values were calculated for each experiment on the basis of four different concentrations using Cheburator software, as described by Nevozhay [60]. The tested compounds at each concentration were tested in triplicates in a single experiment and each experiment was repeated at least three times independently. The results are summarized in Table 2.

### 5.10. Total Platinum Uptake Level

Cultured MCF-7 cells were seeded at a density of 4 × 10^5^ cells/well of culture medium on a 6-well plate (Corning, NY, USA) to a final volume of 2.5 mL. After 48 h of incubation, causing an increase in the number of cells to the value slightly above 1 × 10^6^ cells/well, which was established using a hemocytometer and bright field microscope (Olympus Europe Holding GmbH, Hamburg, Germany), the cells were exposed to tested compounds or cisplatin for 4 h (10 µM the final concentration of each compound). The wells without MCF-7 cells were filled with 1 mL of culture medium-containing compound tested to act as a reference for nonspecific platinum complex adsorption on the surface of the well. After incubation, all wells were washed twice with 2 mL of cold PBS, filled with 400 μL of hot (ca 90 °C) 65% nitric acid, and maintained to stand for 2 h. All wells were finally filled with 1600 μL of water and this solution was collected and analyzed using iCAP 7400 Duo ICP OES instrument (Thermo Fisher Scientific) using the plasma axial view (10 s) at λ = 265.945 nm. The amount of Pt per cell was calculated by subtracting the amount of Pt found in the blank well from the amount of Pt found in the cell-containing wells and normalized to the average number of cells per well. The mean and SD values were calculated on the basis of 4 measurements. The final results are presented in Table 1.

### 5.11. Cell Cycle Analysis

Cultured HL-60 cells (human acute promyelocytic leukemia) were seeded at a density of 2 × 10^5^ cells/mL of culture medium on 24-well plates (Corning, NY, USA) to a final volume of 2 mL. The cells were exposed to compounds at the following concentrations: compound **1**: 20, 40 µg/mL (40, 80 µM, respectively); compound **2**: 0.2, 0.5 µg/mL (0.4, 1 µM, respectively); and cisplatin: 0.1 µg/mL (0.3 µM). The stock solutions of platinum derivatives were prepared as described above. Acetone, used as a solvent for both compounds, was also tested alone exhibiting no cytotoxic effect in 0.1% concentration.

After 48 h of incubation, the cells were collected and washed in phosphate-buffered saline (PBS; HIIET, Wroclaw, Poland) supplemented with 2% of fetal bovine serum and counted in a hemocytometer. Then, the cells (1 × 10^6^ per sample) were washed twice in cold PBS and fixed for 24 h in 70% ethanol at −20 °C. Following this, the cells were washed twice in PBS and incubated with RNAse (8 μg/mL, Fermentas GmbH, St. Leon-Rot, Germany) at 37 °C for 1 h. Finally, the cells were stained for 30 min with propidium iodide (0.5 mg/mL; Sigma-Aldrich, Steinheim, Germany) at 4 °C and the cellular DNA content was determined using a BD LSR Fortessa instrument (Becton Dickinson, San Jose, CA, USA) and WinMDI 2.8 software. The experiment was repeated at least three times.

### 5.12. Determination of Apoptosis by Annexin V Staining

The HL-60 cells and compounds tested were prepared in the same way as for analysis of the cell cycle distribution. The cells were also exposed to camptothecin (2 µg/mL; Sigma-Aldrich, Steinheim, Germany) as a positive control of apoptosis induction. After 48 h of incubation, or in the case of camptothecin after 24 h, the cells were collected and washed in PBS (HIIET, Wroclaw, Poland) supplemented with 2% of fetal bovine serum and counted in a hemacytometer. The cells (2 × 10^5^) were washed twice in PBS. Next, cells were suspended in 200 μL of the mixture of HEPES buffer: 10 mM HEPES, 150 mM NaCl, 5 mM KCl, 1 mM MgCl_2_, 1.8 mM CaCl_2_, (HIIET, Wroclaw, Poland) and Annexin V-FITC (Alexis Biochemicals, San Diego, CA, USA), each time freshly prepared according to the manufacturer’s recommendations. After 15 min of incubation in darkness at room temperature, propidium iodide (PI) solution (0.1 mg/mL) was added prior to analysis to give a final concentration of 10 µg/mL. Data acquisition was performed by flow cytometry on a BD LSR Fortessa instrument (Becton Dickinson, San Jose, CA, USA). The data were displayed as a two-color dot plot with Annexin V-FITC vs. PI. Double-negative cells were live cells, AV+/PI+ were late apoptotic, AV−/PI+ were necrotic cells, and AV+/PI− were early apoptotic cells. Data were analyzed in a BD FACS Diva 6.2 program. The experiment was repeated at least three times [52].

### 5.13. Statistical Analysis

Statistics were performed with Statistica (data analysis software system), version 13.3 (TIBCO Software Inc. (2017), Palo Alto, CA, USA), STATISTICA version 10 (StatSoft, Inc., Tulsa, OK, USA) and PTC Mathcad Express Prime 6.0.0.0. (Copyright 2019. PTC Inc., Boston, MA, U.S.A). For cell cycle and cell death analysis, statistical analysis was performed using GraphPad Prism 7 (GraphPad Software Inc., San Diego, CA, USA). The assumptions of analysis of variance (ANOVA) were checked using the Shapiro–Wilk normality test and Brown–Forsythe test. If the assumptions of the parametric test were found to be fulfilled, one-way ANOVA followed by Dunnett’s multiple comparison test was run. If they were not met, the nonparametric Kruskal–Wallis test was performed followed by Dunn’s multiple comparisons test. The specific tests used for data analysis are listed in the figure legends. Differences with a *p*-value of less than 0.05 were considered to be statistically significant.

## Figures and Tables

**Table 1:** Total Cellular Platinum Uptake in MCF-7 Cells (± SD) (n = 4 or 5) and experimentally measured logP ± SD (n = 4) values.

Compound	ng Pt/10^6^ Cells	logP ± SD
**1**(*cis*), n = 4	27 ± 6 *	−0.35 ± 0.04
**2**(*trans*), n = 5	32 ± 6 *	1.43 ± 0.16,
**3**(*cis*), n = 5	82 ± 11	−0.31 ± 0.04
**4**(*trans*), n = 5	83 ± 12	0.46 ± 0.00
**5**(*cis*), n = 5	39 ± 3 *	−0.58 ± 0.13
**6**(*trans*), n = 4	47 ± 17 *	−0.37 ± 0.03
**7**(*cis*), n = 4	16 ± 7 *	−0.69 ± 0.04
**8**	^†^ below IDL	−1.22 ± 0.08
Cisplatin, n = 4	74 ± 20	−2.05 ± 0.17

* The compounds have been absorbed significantly (*p*-value ≤ 0.05 level probability of statistical significance) weaker than cisplatin by MCF-7 cells. The underlined values have a statistically significant lack of variance homogeneity (Fisher–Snedecor’s *F*-test, *F*_α/2_) with respect to reference drug variance, so Aspin–Welch’s *t*-test was applied in these cases. ^†^ IDL—Instrument Detection Limit.

**Table 2:** Antiproliferative activity of platinum derivatives **1–8** in normoxia and hypoxia conditions against some cancer and normal cell lines. Data are given as Inhibitory Concentration for 50% of tested cells, IC_50_ ± SD [µM].

Compound	Cancer Cells						Normal Cells
	MCF-7		ES-2		A549		BALB/3T3
	Normoxia	Hypoxia	Normoxia	Hypoxia	Normoxia	Hypoxia	Normoxia
**1**(*cis*)	74.7 ± 36.1*	inactive	29.4 ± **24.9**	108.7 ± 22.6*	103.3 ± 28.8*	inactive	>100 *
**2**(*trans*)	1.8 ± 0.9 **	11.1 ± 5.6	0.7 ± 0.7 **	1.1 ± **0.3** **	3.7 ± 2.9 **	7.7 ± 7.4 *	8.19 ± 2.9
**3**(*cis*)	10.1 ± 1.4	41.5 ± **39.9**	75.4 ± 12.3*	51.9 ± 16.7 *	53.4 ± 10.0*	inactive	>100 *
**4**(*trans*)	7.8 ± 1.3 **	32.0 ± 9.2 *	41.1 ± 7.1 *	32.3 ± **27.0**	31.0 ± 9.5*	inactive	74.4 ± **23.2** *
**5**(*cis*)	49.1 ± **30.1**	inactive	38.1 ± 9.2 *	inactive	inactive	inactive	>100 *
**6**(*trans*)	23.6 ± 8.6	86.8 ± 19.0 *	36.3 ± 8.6 *	99.8 ± 37.9*	58.2 ± 7.6*	inactive	>100 *
**7**(*cis*)	58.0 ± 8.7 *	inactive	68.9 ± 10.0 *	inactive	93.0 ± 12.9*	inactive	>100 *
**8** *(ionic)*	inactive	inactive	48.5 ± **25.9**	inactive	inactive	inactive	>100 *
1-Methyl-4-nitropyrazole	inactive	inactive	inactive	inactive	inactive	inactive	>100 *
Cisplatin	12.6 ± 2.6	14.7 ± 5.8	8.6 ± 2.6	13.7 ± 5.7	9.8 ± 1.2	23.9± 9.3	8.67± 2.6

* less cytotoxic than cisplatin, ** more cytotoxic than cisplatin—The difference between the mean value of tested compound cytotoxicity and the mean value of cytotoxicity of reference cisplatin is statistically significant at the α = 0.05 threshold of significance level (*p*-value ≤ 0.05 of probability level of statistical significance, Student’s *t*-test (n = 3 for normoxia, n = 4 for hypoxia)). However, the averages with the bolded values of SD have a statistically significant lack of variance homogeneity (Fisher–Snedecor’s *F*-test, *F*_α/2_) with respect to reference drug variance, so Aspin–Welch’s *t*-test was applied in these cases. The underlined values show a statistically significant difference between cytotoxic activity in normoxia and hypoxia condition.

**Table 3:** Selected X-ray Data for di-acetone solvate of Compound **2**.

Compound No	2
**Formula**	**C_14_H_22_Cl_2_N_6_O_6_Pt**
Formula weight	636.36
Temperature [K]	100(2)
λ [Å]	0.71073
Crystal system	Orthorhombic
Space group	Pbcn (No.60)
a [Å]	22.0311(7)
b [Å]	12.2362(4)
c [Å]	8.1475(3)
α [°]	
β [°]	
γ [°]	
V [Å^3^]	2196.38(13)
Z, ρ_calc_ [g cm^−3^]	4, 1.924
μ [mm^−1^]	6.673
F(000)	1232
Crystal size [mm]	0.17 × 0.10 × 0.03
θ range[°]	3.235 to 28.891°.
rflns: total/unique	7142/2571
Abs. corr.	analytical
Min., max. transmission factors	0.897/0.789
Data/restraints/params	2571/0/136
GOF on F^2^	1.033
R_1_ [I > 2σ(I)]	0.0263
wR_2_ (all data)	0.0542
Max., min. Δρ_elect_ [e Å^3^]	1011/−0.870

CCDC reference number for compound **2**: CCDC2044576 (supplementary data available from CCDC, 12 Union Road, Cambridge CB2, 1EZ, UK on request).

## Supplementary Materials

The following are available online at https://www.mdpi.com/1424-8247/13/12/433/s1, CCDC reference number for compound **1**: CCDC924455; for compound **2**: CCDC2044576—these data can be obtained on request free of charge via http://www.ccdc.cam.ac.uk/getstructures (or from the Cambridge Crystallographic Data Centre, 12, Union Road, Cambridge CB2 1EZ, U.K.; Fax: +44-1223-336033). Besides, the supplementary data and additional information associated with this paper can be found online as the Supplementary Material file (pdf) which contains, among others, the visualizations of ESI-MS, IR and NMR spectra of the considered compounds. Crystallographic Information File (CIF) representing crystallographic information of the compound **2** is also attached.

## References

[1] Montaña Á.M., Batalla C. (2009). The Rational Design of Anticancer Platinum Complexes: The Importance of the Structure-Activity Relationship. Curr. Med. Chem..

[2] Chen Y., Hu L. (2009). Design of Anticancer Prodrugs for Reductive Activation. Med. Res. Rev..

[3] Mistry I.N., Thomas M., Calder E.D.D., Conway S.J., Hammond E.H. (2017). Clinical Advances of Hypoxia-Activated Prodrugs in Combination With Radiation Therapy. Int. J. Radiat. Oncol. Biol. Phys..

[4] Gilmour D.W., Sadler P.J. (1984). New Metal Complexes of 4-Nitrosubstituted Pyrazoles, Imidazoles and Isothiazoles. GB(UK) Patent.

[5] Cleare M.J., Hoeschele J.D. (1973). Antitumor platinum compounds. Relationship between structure and activity. Platin. Met. Rev..

[6] Reedijk J. (2011). Increased understanding of platinum anticancer chemistry. Pure Appl. Chem..

[7] Cleare M.J., Hoeschele J.D. (1973). Studies on the antitumor activity of group VIII transition metal complexes. Part I. Platinum (II) complexes. Bioinorg. Chem..

[8] Cai L., Yu C., Ba L., Liu Q., Quian Y., Yang B., Gao C. (2018). Anticancer platinum based complexes with nonclassical structures. Appl. Organomet. Chem..

[9] Brabec V., Hrabina O., Kasparkova J. (2017). Cytotoxic platinum coordination compounds. DNA binding agents; Coord. Chem. Rev..

[10] Quiroga A.G. (2011). Non-Classical Structures among Current Platinum Complexes with Potential as Antitumor Drugs. Curr. Top. Med. Chem..

[11] Rakić G.M., Grgurić-Sĭpka S., Kaluđerović G.N., Gómez-Ruiz S., Bjelogrlić S.K., Radulović S.S., Tešić Z.L. (2009). Novel trans-dichloridoplatinum(II) complexes with 3- and 4-acetylpyridine: Synthesis, characterization, DFT calculations and cytotoxicity. Eur. J. Med. Chem..

[12] Filipovic L., Arandelovic S., Gligorijevic N., Krivokuca A., Jankovic R., Srdic-Rajic T., Rakic G., Tesic Z., Radulovic S. (2013). Biological evaluation of transdichloridoplatinum(II) complexes with 3- and 4-acetylpyridine in comparison to cisplatin. Radiol. Oncol..

[13] Farrell N., Ha T.T.B., Souchard J.P., Wimmer F.L., Cros S., Johnson N.P. (1989). Cytostatic trans-platinum(II) Complexes. J. Med. Chem..

[14] Natile G., Coluccia M. (2001). Current status of trans-platinum compounds in cancer therapy. Coord. Chem. Rev..

[15] Coluccia M., Natile G. (2007). Trans-Platinum Complexes in Cancer Therapy. Anti Cancer Agents Med. Chem..

[16] Knipp M., Karotki A.V., Chesnov S., Natile G., Sadler P.J., Brabec V., Vašak M. (2007). Reaction of Zn_7_Metallothionein with cis- and trans-Pt(N-donor)_2_Cl_2_ Anticancer Complexes: Trans-Pt^II^ Complexes Retain Their N.-Donor Ligands. J. Med. Chem..

[17] Li C., Li Z., Sletten E., Arnesano F., Losacco M., Natile G., Liu Y. (2009). Methionine Can Favor DNA Platination by trans-Coordinated Platinum Antitumor Drugs. Angew. Chem. Int. Ed..

[18] Xu D., Min Y., Cheng Q., Shi H., Wei K., Arnesano F., Natile G., Liu Y. (2013). Chemical and cellular investigations of trans-ammine-pyridinedichlorido-platinum(II), the likely metabolite of the antitumor active cis-diammine-pyridine-chorido-platinum(II), Pt(IV) derivatives. J. Inorg. Biochem..

[19] McGowan G., Parsons S., Sadler P.J. (2005). Contrasting Chemistry of cis- and trans-Platinum(II) Diamine Anticancer Compounds: Hydrolysis Studies of Picoline Complexes. Inorg. Chem..

[20] Fabijańska M., Orzechowska M., Rybarczyk-Pirek A.J., Dominikowska J., Bieńkowska A., Małecki M., Ochocki J. (2020). Simple Trans-Platinum Complex Bearing 3-Aminoflavone Ligand Could Be a Useful Drug: Structure-Activity Relationship of Platinum Complex in Comparison with Cisplatin. Int. J. Mol. Sci..

[21] Kelland L.R., Abel G., McKeage M.J., Jones M., Goddard P.M., Valenti M., Murrer B.A., Harrap K.R. (1993). Preclinical antitumor evaluation of bis-acetato-ammine-dichloro-cyclohexylamine platinum(IV): An orally active platinum drug. Cancer Res..

[22] Johnstone T.C., Suntharalingam K., Lippard S.J. (2016). The Next Generation of Platinum Drugs: Targeted Pt(II) Agents, Nanoparticle Delivery, and Pt(IV) Prodrugs. Chem. Rev..

[23] Mangrum J.B., Farrell N.P. (2010). Excursions in polynuclear platinum DNA binding. Chem. Commun..

[24] Farrell N.P. (2015). Multi-platinum Anti-cancer Agents. Substitution-inert Compounds for Tumor Selectivity and New Targets. Chem. Soc. Rev..

[25] Raynaud F.I., Boxall F.E., Goddard P.M., Valenti M., Jones M., Murrer B.A., Abrams M., Kelland L.R. (1997). cis-Amminedichloro(2-methylpyridine) platinum(II) (AMD473), a novel sterically hindered platinum complex: In vivo activity, toxicology, and pharmacokinetics in mice. Clin. Cancer Res..

[26] Mugge C., Rothenburger C., Beyer A., Görls H., Gabbiani C., Casini A., Michelucci E., Landini I., Nobili S., Mini E. (2011). Structure, solution chemistry, antiproliferative actions and protein binding properties of non-conventional platinum(II) compounds with sulfur and phosphorus donors. Dalton Trans..

[27] Ma E.S.F., Bates W.D., Edmunds V., .Kelland L.R., Fojo T., Farrell N. (2005). Enhancement of aqueous solubility and stability employing a trans acetate axis in trans planar amine platinum compounds while maintaining the biological profile. J. Med. Chem..

[28] Wheate N.J., Walker S., Craig G.E., Oun R. (2010). The status of platinum anticancer drugs in the clinic and in clinical trials. Dalton Trans..

[29] Bhargava A., Vaishampayan U.N. (2009). Satraplatin: Leading the new generation of oral platinum agents. Expert Opin. Investig. Drugs.

[30] Skov K.A., Farrell N.P., Chaplin D.J. (1990). Platinum Complexes with One Radiosensitizing Ligand. U.S. Patent.

[31] Brown J.M. (1999). The hypoxic cell: A target for selective cancer therapy—Eighteenth Bruce F. Cain memorial award lecture. Cancer Res..

[32] Regiec A., Wojciechowski P., Mastalarz H. (2014). Experimental and theoretical spectroscopic and electronic properties enriched with NBO analysis for 1-methyl-3-nitropyrazole and 1-methyl-5-nitropyrazole. J. Mol. Struct..

[33] Regiec A., Mastalarz H., Wojciechowski P. (2014). Theoretical anharmonic Raman and infrared spectra with vibrational assignments and NBO analysis for 1-methyl-4-nitropyrazole. J. Mol. Struct..

[34] Regiec A., Wojciechowski P. (2019). Synthesis and experimental versus theoretical research on spectroscopic and electronic properties of 3-methyl-4-nitroisothiazole. J. Mol. Struct..

[35] Katritzky A.R., Scriven E.F.V., Majumder S., Akhmedova R.G., Akhmedov N.G., Vakulenko A.V. (2005). Direct nitration of five membered heterocycles. ARKIVOC.

[36] Roy S., Hagen K.D., Maheswari P.U., Lutz M., Spek A.L., Reedijk J., van Wezel G.P. (2008). Phenanthroline derivatives with improved selectivity as DNA-targeting anticancer or antimicrobial drugs. ChemMedChem.

[37] Marqués-Gallego P., Kalayda G.V., Jaehde U., den Dulk H., Brouwer J., Reedijk J. (2008). Cellular accumulation and DNA platination of two new platinum(II) anticancer compounds based on anthracene derivatives as carrier ligands. Inorg. Chem..

[38] Tocher J.H. (1997). Reductive activation of nitroheterocyclic compounds. Gen. Pharmacol..

[39] Sisson G., Goodwin A., Raudonikiene A., Hughes N.J., Mukhopadhyay A.K., Berg D.E., Hoffman P.S. (2002). Enzymes Associated with Reductive Activation and Action of Nitazoxanide, Nitrofurans, and Metronidazole in Helicobacter pylori. Antimicrob. Agents Chemother.

[40] Zięba-Mizgała A., Puszko A., Regiec A., Kuduk-Jaworska J. (2005). Electrophilic properties of nitroheterocyclic compounds. Potential hypoxic cells radiosensitizers. Bioelectrochemistry.

[41] Papesch V., Dodson R.M. (1965). Isomeric Pyrazolo[4,3-d]pyrimidinedione. J. Org. Chem..

[42] Regiec A., Mastalarz H., Mastalarz A., Kochel A. (2009). Methylation of 4-nitro-3(5)-pyrazolecarboxylic acid. Tetrahedron Lett..

[43] Regiec A., Mastalarz A., Wietrzyk J., Mastalarz H. (2016). Cis-and trans-Platinum Complex Compounds (II) with 1-Methyl-4-Nitropyrazole, the Process for Their Preparation, Separation, Isomerization, and the Use for the Manufacture of Medicaments for Tumor Therapy. Polish Patent.

[44] Allen A.D., Theophanides T. (1964). Platinum(II) Complexes: Infrared Spectra In The 300–800 cm^−1^ Region. Can. J. Chem..

[45] Tetko I.V., Jaroszewicz I., Platts J.A., Kuduk-Jaworska J. (2008). Calculation of lipophilicity for Pt(II) complexes: Experimental comparison of several methods. J. Inorg. Biochem..

[46] Reithofer M.R., Valiahdi S.M., Galanski M., Jakupec M.A., Arion V.B., Keppler B.K. (2008). Novel endothall containing platinum(IV) complexes-synthesis, characterization, and cytotoxic activity. Chem. Biodivers..

[47] Wilson J.J., Lippard S.J. (2012). In Vitro Anticancer Activity of *cis*-Diammineplatinum(II) Complexes with β-Diketonate Leaving Group Ligands. J. Med. Chem..

[48] Jamalzadeh L., Ghafoori H., Sariri R., Rabuti H., Nasirzade J., Hasani H., Aghamaali M.R. (2016). Cytotoxic Effects of Some Common Organic Solvents on MCF-7, RAW-264.7 and Human Umbilical Vein Endothelial Cells. Avicenna J. Med. Biochem..

[49] Johnstone T.C., Wilson J.J., Lippard S.J. (2013). Monofunctional and Higher-Valent Platinum Anticaner. Inorg. Chem..

[50] Cherian M.G. (1994). The Significance of the Nuclear and Cytoplasmic Localization of Metallothionein in Human Liver and Tumor Cells. Environ. Health Perspect..

[51] Hagrman D., Godisman J., Dabrowiak J.C., Souild A.K. (2003). Kinetic study on the reaction of cisplatin with metallothionein. Drug Metab. Dispos..

[52] Vermes I., Haanen C., Steffens-Nakken H., Reutelingsperger C. (1995). A novel assay for apoptosis. Flow cytometric detection of phosphatidylserine expression on early apoptotic cells using fluorescein labelled Annexin V. J. Immunol. Methods.

[53] Lecoeur H. (2002). Nuclear apoptosis detection by flow cy-tometry: Influence of endogenous endonucleases. Exp. Cell Res..

[54] Suchankova T., Vojtıskova M., Reedijk J., Brabec V., Kasparkova J. (2009). DNA and glutathione interactions in cell-free media of asymmetric platinum(II) complexes cis- and trans-[PtCl2(isopropylamine) (1-methylimidazole)]: Relations to their different antitumor effects. J. Biol. Inorg. Chem..

[55] Gałczyńska K., Drulis-Kawa Z., Arabski M. (2020). Antitumor Activity of Pt(II), Ru(III) and Cu(II) Complexes. Molecules.

[56] Rigaku O.D. (2017). CrysAlis PRO.

[57] Sheldrick G.M. (2015). Crystal structure refinement with SHELXL. Acta Crystallogr. Sect. C Struct. Chem..

[58] Putz H., Brandenburg K. Diamond-Crystal and Molecular Structure Visualization.

[59] Skehan P., Storeng R., Scudiero D., Monks A., McMahon J., Vistica D., Warren J.T., Bokesch H., Kenney S., Boyd M.R. (1990). New Colorimetric Cytotoxicity Assay for Anticancer-Drug Screening. J. Natl. Cancer Inst..

[60] Nevozhay D. (2014). Cheburator software for automatically calculating drug inhibitory concentrations from in vitro screening assays. PLoS ONE.

