# Supplementary material for: Synthesis of Platinum(II) Complexes with Some 1-Methylnitropyrazoles and In Vitro Research on Their Cytotoxic Activity
Source: Pharmaceuticals (Basel). 2020 Nov 28;13(12):433. doi: 10.3390/ph13120433 (PMC7768359; doi:10.3390/ph13120433)
Supplement: Supplementary file 1 [file pharmaceuticals-13-00433-s001.zip › checkCIFtransPT.pdf]

checkCIF (basic structural check) running

Checking for embedded fcf data in CIF ...
Found embedded fcf data in CIF. Extracting fcf data from uploaded CIF, please wait . .

checkCIF/PLATON (basic structural check)

Structure factors have been supplied for datablock(s) IG3

THIS REPORT IS FOR GUIDANCE ONLY. IF USED AS PART OF A REVIEW PROCEDURE FOR PUBLICATION, IT SHOULD NOT REPLACE THE EXPERTISE OF AN EXPERIENCED CRYSTALLOGRAPHIC REFEREE.

No syntax errors found. CIF dictionary
Please wait while processing .... Interpreting this report
Structure factor report

Datablock: IG3

```
Bond precision:      C-C = 0.0055 A            Wavelength=0.71073
Cell:          a=22.0311(7)   b=12.2362(4)   c=8.1475(3)
               alpha=90       beta=90        gamma=90
Temperature: 100 K
```

	Calculated	Reported
Volume	2196.38(13)	2196.38(13)
Space group	P b c n	P b c n
Hall group	-P 2n 2ab	-P 2n 2ab
Moiety formula	C8 H10 Cl2 N6 O4 Pt, 2(C3 H6 O)	C8 H10 Cl2 N6 O4 Pt, 2(C3 H6 O)
Sum formula	C14 H22 Cl2 N6 O6 Pt	C14 H22 Cl2 N6 O6 Pt
Mr	636.36	636.36
Dx,g cm-3	1.924	1.924
Z	4	4
Mu (mm-1)	6.673	6.673
F000	1232.0	1232.0
F000'	1227.09	
h,k,lmax	29,16,11	29,16,10
Nref	2881	2571
Tmin,Tmax	0.453,0.819	0.789,0.897
Tmin'	0.318	

```
Correction method= # Reported T Limits: Tmin=0.789
Tmax=0.897 AbsCorr = ANALYTICAL
Data completeness= 0.892        Theta(max)= 28.891
R(reflections)= 0.0263( 1810)      wR2(reflections)= 0.0542( 2571)
S = 1.033               Npar= 136
```

The following ALERTS were generated. Each ALERT has the format
test-name_ALERT_alert-type_alert-level.
Click on the hyperlinks for more details of the test.

Alert level C

PLAT906_ALERT_3_C Large K Value in the Analysis of Variance ...... 6.170 Check
PLAT906_ALERT_3_C Large K Value in the Analysis of Variance ...... 2.170 Check

Alert level G

PLAT432_ALERT_2_G Short Inter X...Y Contact O006 ..C00D 2.98 Ang.
x,1-y,1/2+z = 7_566 Check
PLAT720_ALERT_4_G Number of Unusual/Non-Standard Labels .... 27 Note
PLAT794_ALERT_5_G Tentative Bond Valency for Pt01 (II) . 2.22 Info
PLAT883_ALERT_1_G No Info/Value for _atom_sites_solution_primary . Please Do !
PLAT910_ALERT_3_G Missing # of FCF Reflection(s) Below Theta(Min). 3 Note
PLAT912_ALERT_4_G Missing # of FCF Reflections Above STh/L= 0.600 305 Note
PLAT941_ALERT_3_G Average HKL Measurement Multiplicity .... 2.8 Low
PLAT978_ALERT_2_G Number C-C Bonds with Positive Residual Density. 1 Info

0 **ALERT level A** = Most likely a serious problem - resolve or explain
0 **ALERT level B** = A potentially serious problem, consider carefully
2 **ALERT level C** = Check. Ensure it is not caused by an omission or oversight
8 **ALERT level G** = General information/check it is not something unexpected

1 ALERT type 1 CIF construction/syntax error, inconsistent or missing data
2 ALERT type 2 Indicator that the structure model may be wrong or deficient
4 ALERT type 3 Indicator that the structure quality may be low
2 ALERT type 4 Improvement, methodology, query or suggestion
1 ALERT type 5 Informative message, check

It is advisable to attempt to resolve as many as possible of the alerts in all categories. Often the minor alerts point to easily fixed oversights, errors and omissions in your CIF or refinement strategy, so attention to these fine details can be worthwhile. In order to resolve some of the more serious problems it may be necessary to carry out additional measurements or structure refinements. However, the purpose of your study may justify the reported deviations and the more serious of these should normally be commented upon in the discussion or experimental section of a paper or in the "special_details" fields of the CIF. checkCIF was carefully designed to identify outliers and unusual parameters, but every test has its limitations and alerts that are not important in a particular case may appear. Conversely, the absence of alerts does not guarantee there are no aspects of the results needing attention. It is up to the individual to critically assess their own results and, if necessary, seek expert

advice.

Publication of your CIF in IUCr journals

A basic structural check has been run on your CIF. These basic checks will be run on all CIFs submitted for publication in IUCr journals (*Acta Crystallographica*, *Journal of Applied Crystallography*, *Journal of Synchrotron Radiation*); however, if you intend to submit to *Acta Crystallographica Section C* or *E* or *IUCrData*, you should make sure that full publication checks are run on the final version of your CIF prior to submission.

Publication of your CIF in other journals

Please refer to the *Notes for Authors* of the relevant journal for any special instructions relating to CIF submission.

PLATON version of 18/09/2020; check.def file version of 20/08/2020

Datablock IG3 - ellipsoid plot

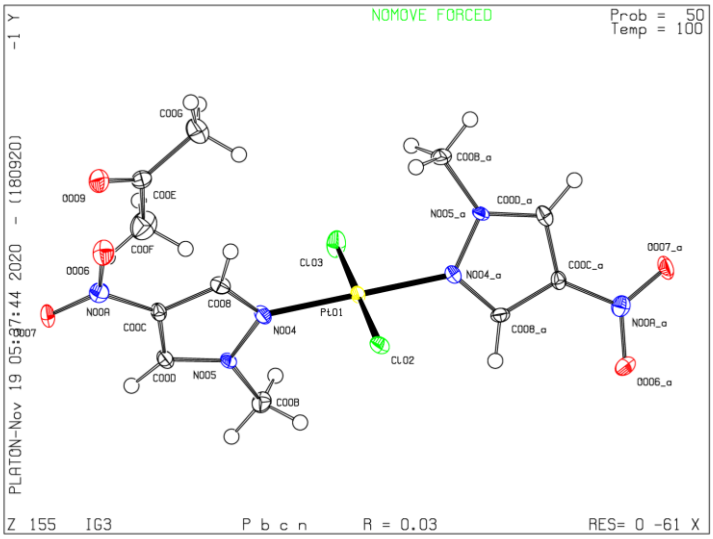

Download CIF editor (publCIF) from the IUCr
Download CIF editor (enCIFer) from the CCDC
Test a new CIF entry
