# Supplementary material for: Synthesis of Platinum(II) Complexes with Some 1-Methylnitropyrazoles and In Vitro Research on Their Cytotoxic Activity
Source: Pharmaceuticals (Basel). 2020 Nov 28;13(12):433. doi: 10.3390/ph13120433 (PMC7768359; doi:10.3390/ph13120433)
Supplement: Supplementary file 1 [file pharmaceuticals-13-00433-s001.zip › Supplementary_Material_ultimate.pdf]

SUPPLEMENTARY MATERIAL

Synthesis of platinum(II) complexes with some 1-methylnitropyrazoles and *in vitro* research on their cytotoxic activity

Henryk Mastalarz[1*], Agnieszka Mastalarz[2], Joanna Wietrzyk[3], Magdalena Milczarek[3], Andrzej Kochel[2] and Andrzej Regiec[1]

[1] Department of Organic Chemistry, Faculty of Pharmacy, Wrocław Medical University,

211A Borowska Street, 50-556 Wrocław, Poland

[2] Faculty of Chemistry, Wrocław University, 14F Joliot-Curie Street, 50-383 Wrocław, Poland

[3] Hirszfeld Institute of Immunology and Experimental Therapy Polish Academy of Sciences, 12 Rudolf Weigl Street, 53-114 Wrocław, Poland

Table of Contents

Simulated ESI MS spectra of compounds **1** and **2 (Fig. S1-4)**	p. **2**
Experimental ESI MS spectra of compounds **1-8 (Fig. S5-12)**	pp **3-7**
Experimental far IR spectra of compounds **1-8 (Fig. S13-20)**	pp **8-11**
Experimental ^{1}H and ^{195}Pt NMR spectra of compounds **1- 2 (Fig. S21, S22)**	p. **12**
Experimental ^{1}H NMR spectra of compounds **3-8 (Fig. S23-26)**	pp **13-16**
Molecular crystallographic structure of compound **1 (FigS27)**	p. **16**
Selected X-ray data for compound **1** (**Table 5**)	p. **17**

*Corresponding author, e-mail: Henryk Mastalarz (henryk.mastalarz@umed.wroc.pl)

Simulated (Fig. S1-3) and experimental (Fig. S5 -12) molecular and isotope peaks in ESI-MS spectrum for compound **1** and **2**. Simulation performed with Compass DataAnalysis Software version. 4.2 (copyright 1993-2003 Bruker Daltonik GmbH).

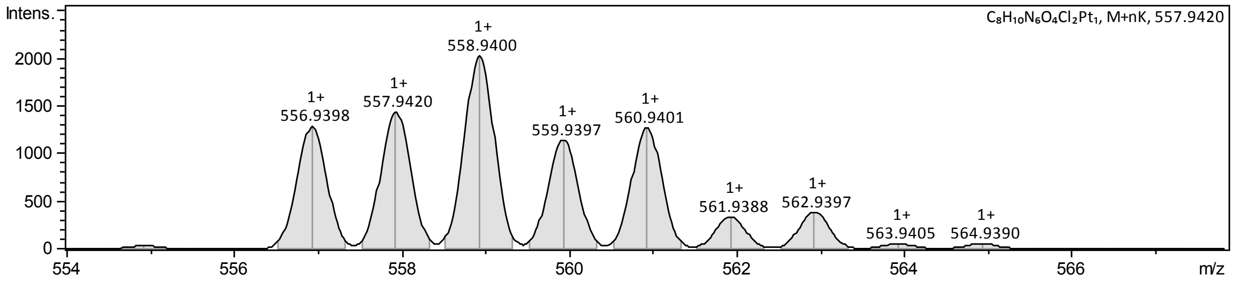

Fig.S1. Simulated quasi molecular (557.9420 Da) and isotope peaks for formula $C_8H_{10}N_6O_4Cl_2Pt+K^+$ in ESI-MS (positive ionization) spectrum

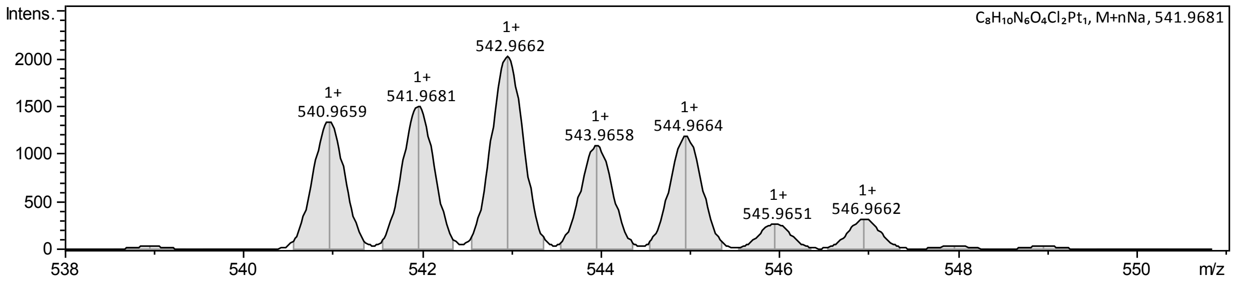

Fig.S2. Simulated quasi molecular (541.9681 Da) and isotope peaks formula $C_8H_{10}N_6O_4Cl_2Pt+Na^+$ in ESI-MS (positive ionization)

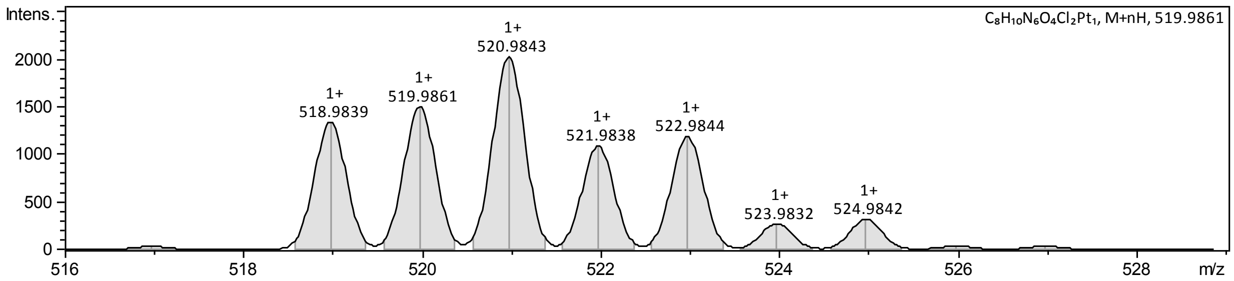

Fig.S3. Simulated quasi molecular (519.9861 Da) and isotope peaks for formula $C_8H_{10}N_6O_4Cl_2Pt+H^+$ in ESI-MS (positive ionization)

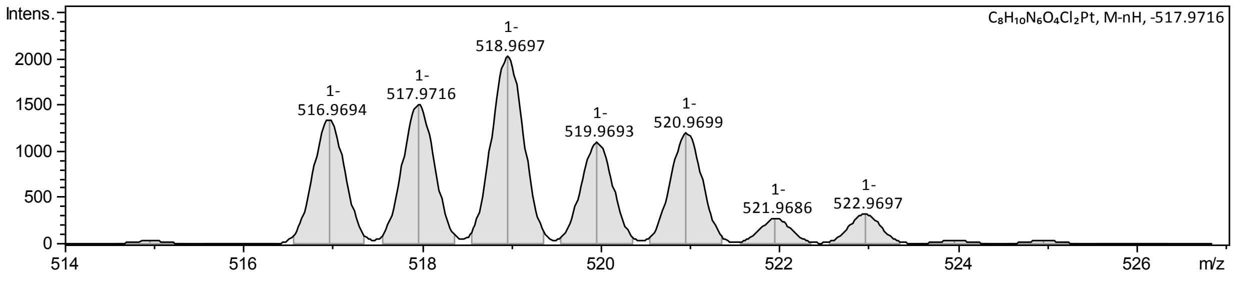

Fig.S4. Simulated quasi molecular (517.9716 Da) and isotope peaks for formula $[C_8H_{10}N_6O_4Cl_2Pt-H]^-$ in ESI-MS (negative ionization)

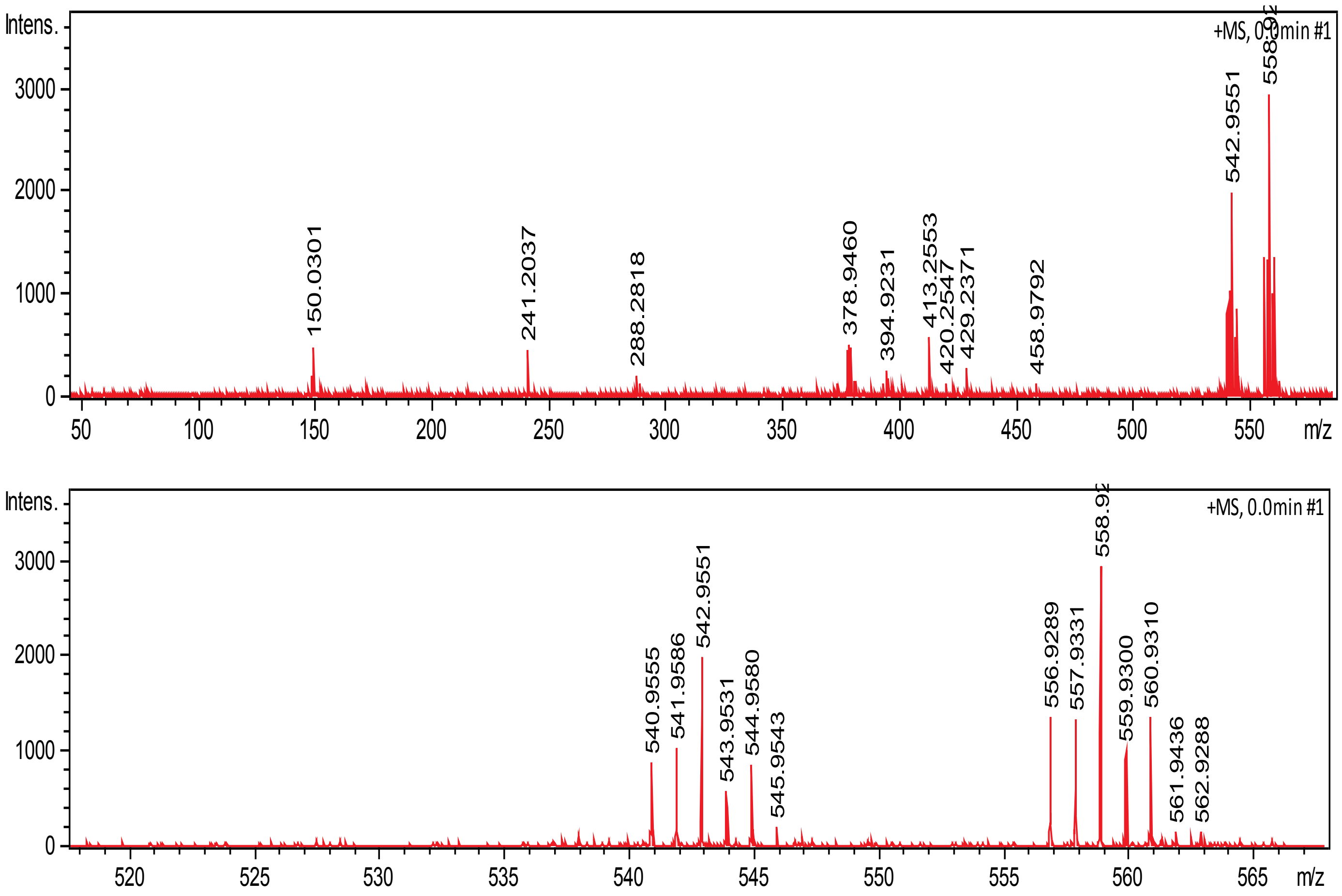

Fig.S5. Experimental ESI-MS (positive ionization in MeOH) spectrum of the compounds **1** and **2**. Quasi molecular and isotope peaks for formula $C_8H_{10}N_6O_4Cl_2Pt+Na^+$ (541.9586 Da) and for formula $C_8H_{10}N_6O_4Cl_2Pt+K^+$ (557.9331 Da).

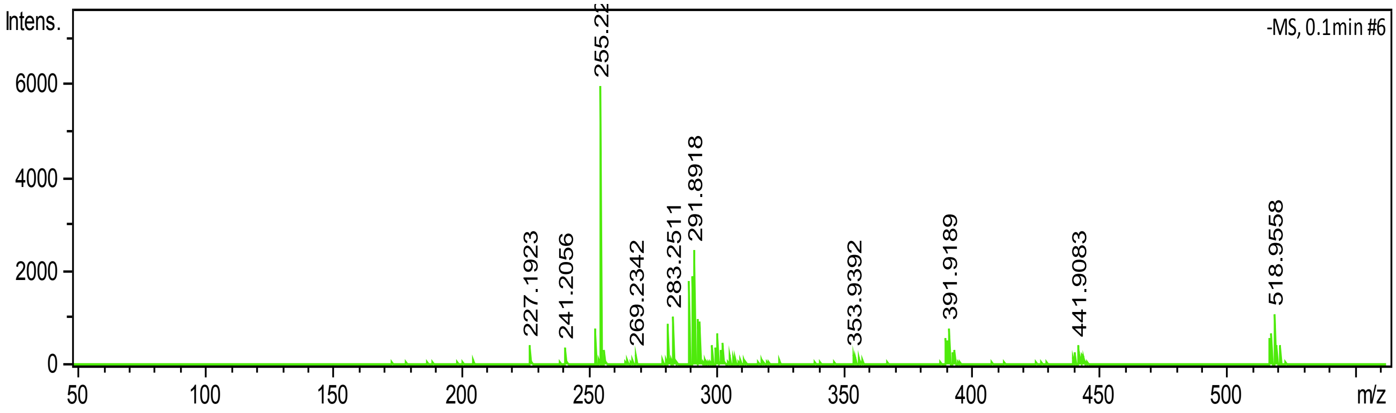

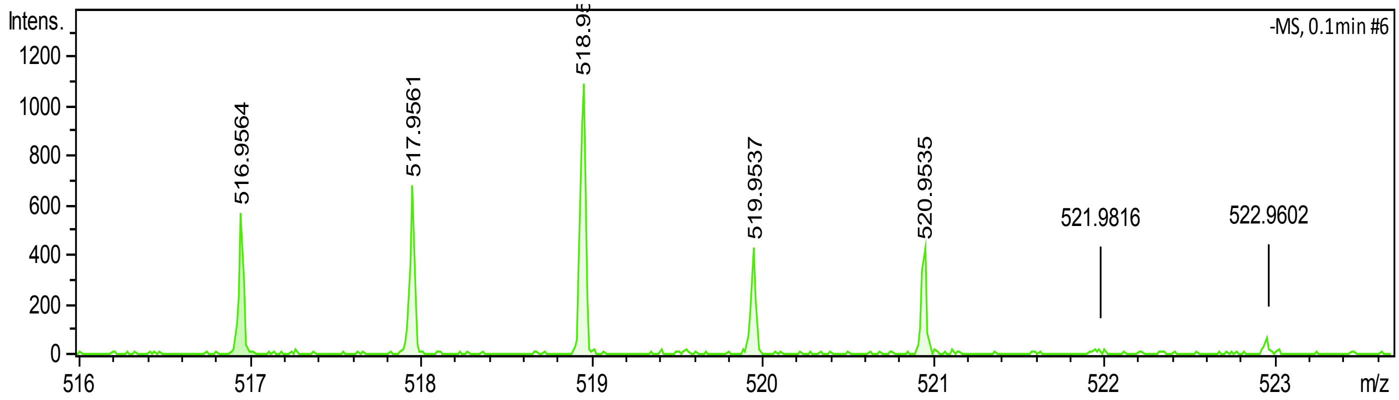

Fig.S6. Experimental ESI-MS (negative ionization in MeOH) spectrum of the compounds **1** and **2**. Quasi molecular and isotope peaks for formula $[C_8H_{10}N_6O_4Cl_2Pt\text{-}H]^-$ (517.9561 Da).

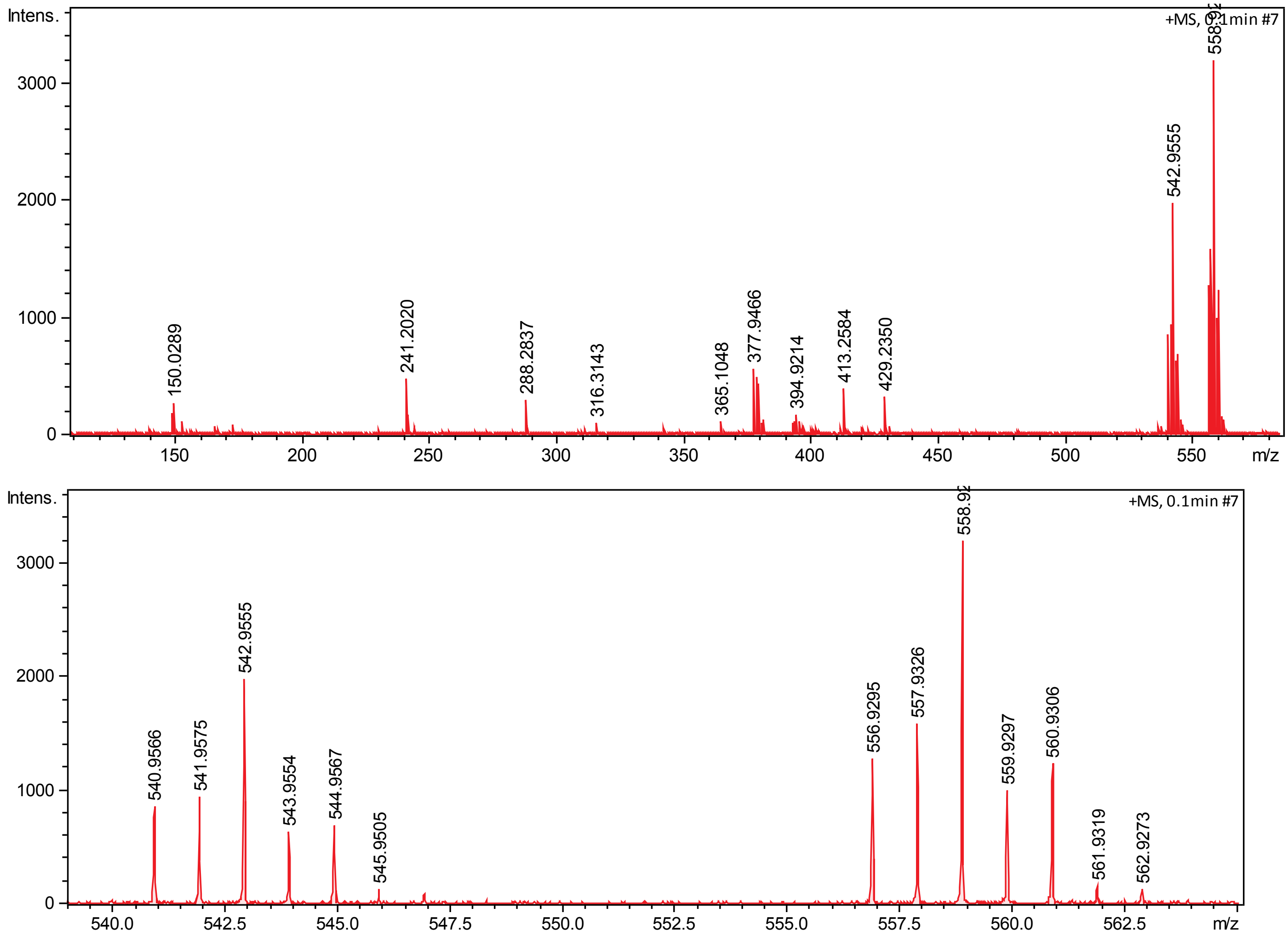

Fig.S7 Experimental ESI-MS (positive ionization in MeOH) spectrum of the compounds **3** and **4**

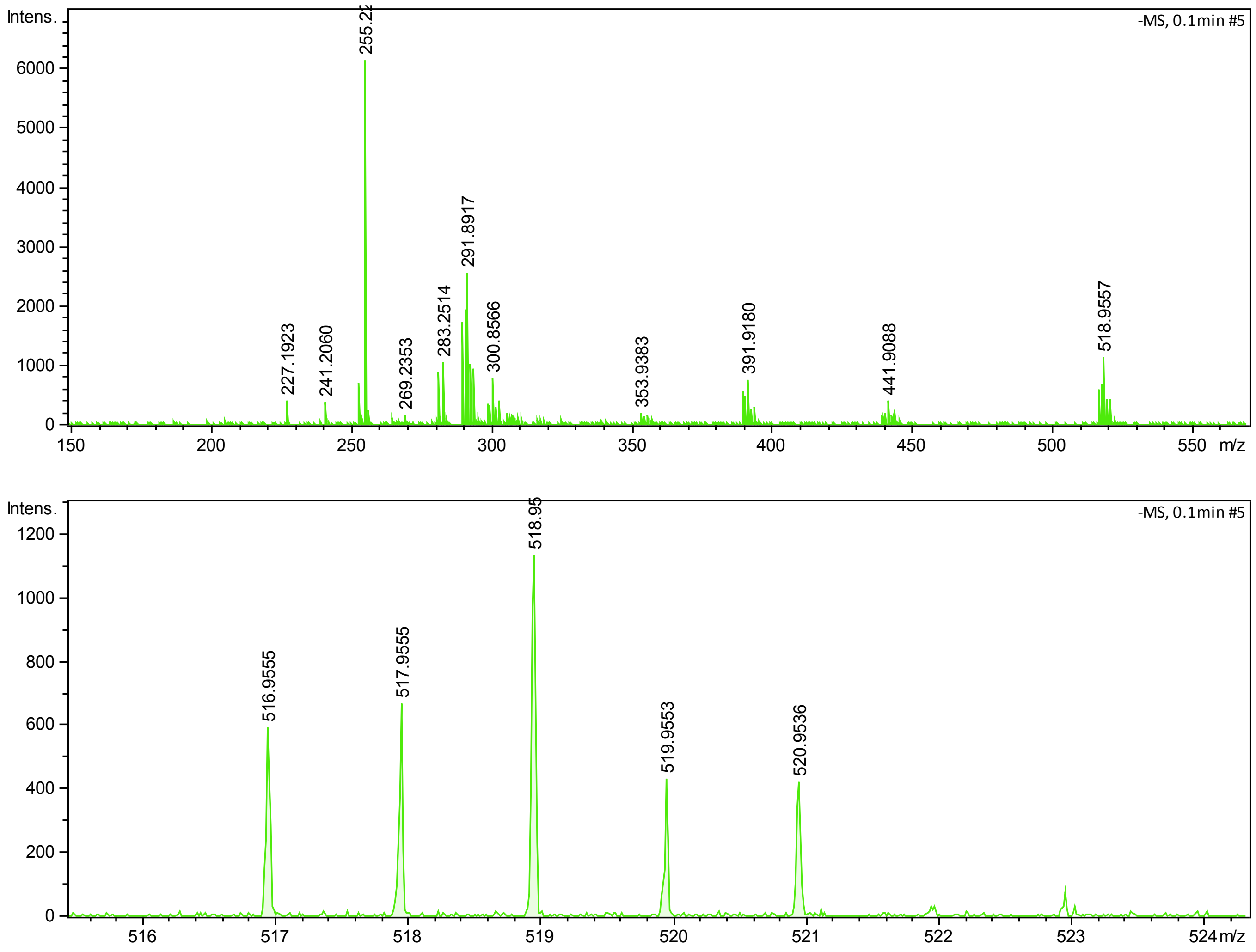

Fig.S8 Experimental ESI-MS (negative ionization in MeOH) spectrum of the compounds **3** and **4**

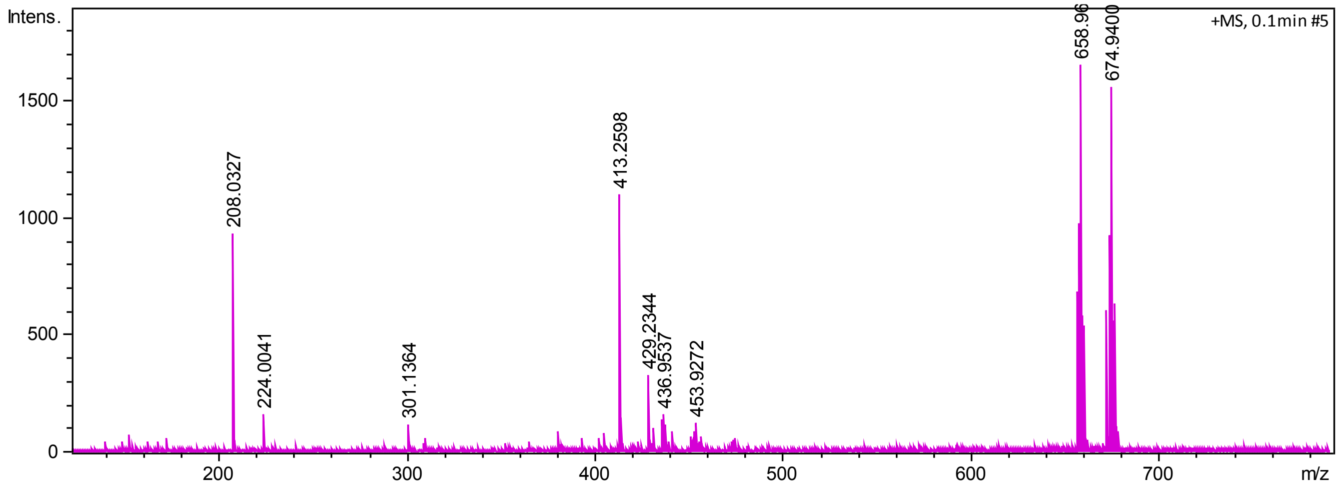

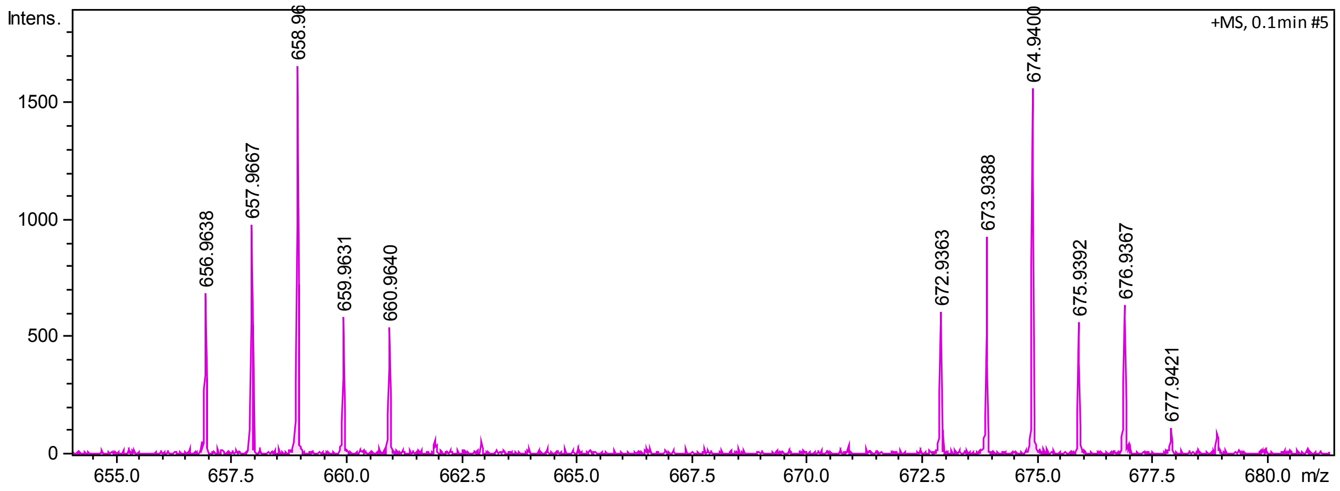

Fig.S9 Experimental ESI-MS (positive ionization in MeOH) spectrum of the compounds **5** and **6**

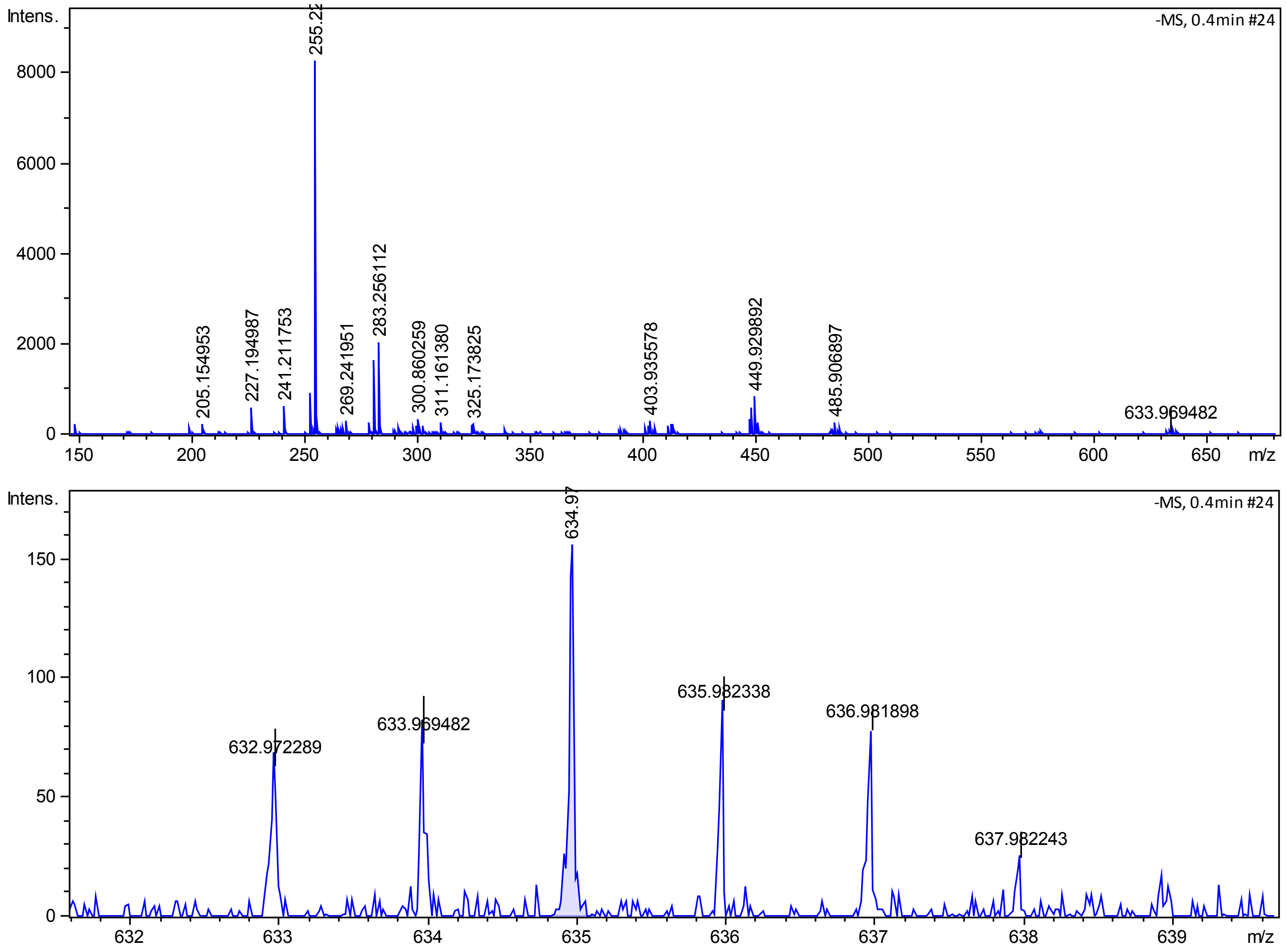

Fig.S10 Experimental ESI-MS (negative ionization in MeOH) spectrum of the compounds **5** and **6**

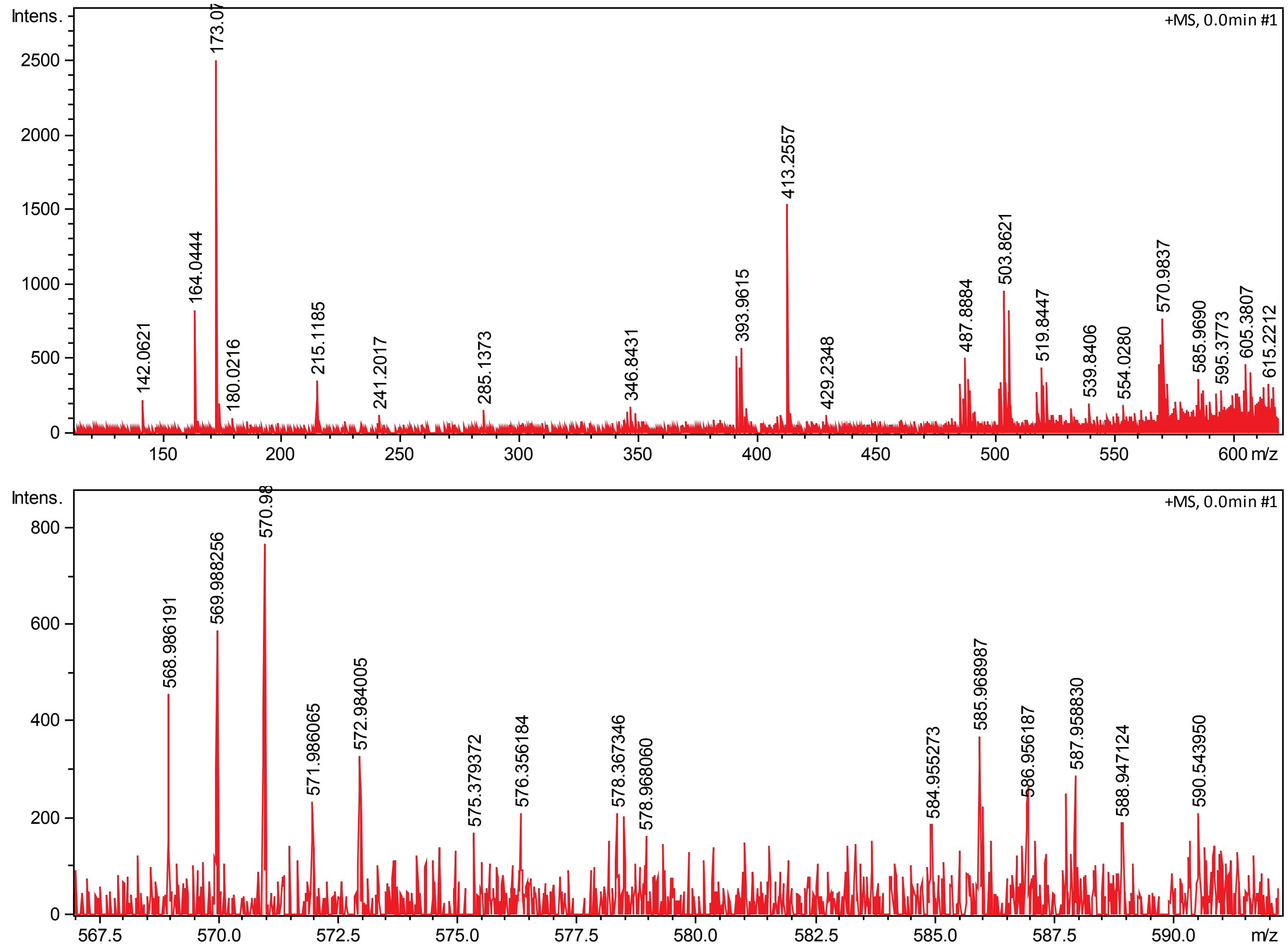

Fig.S11 Experimental ESI-MS (positive ionization in MeOH) spectrum of the compound **7**.

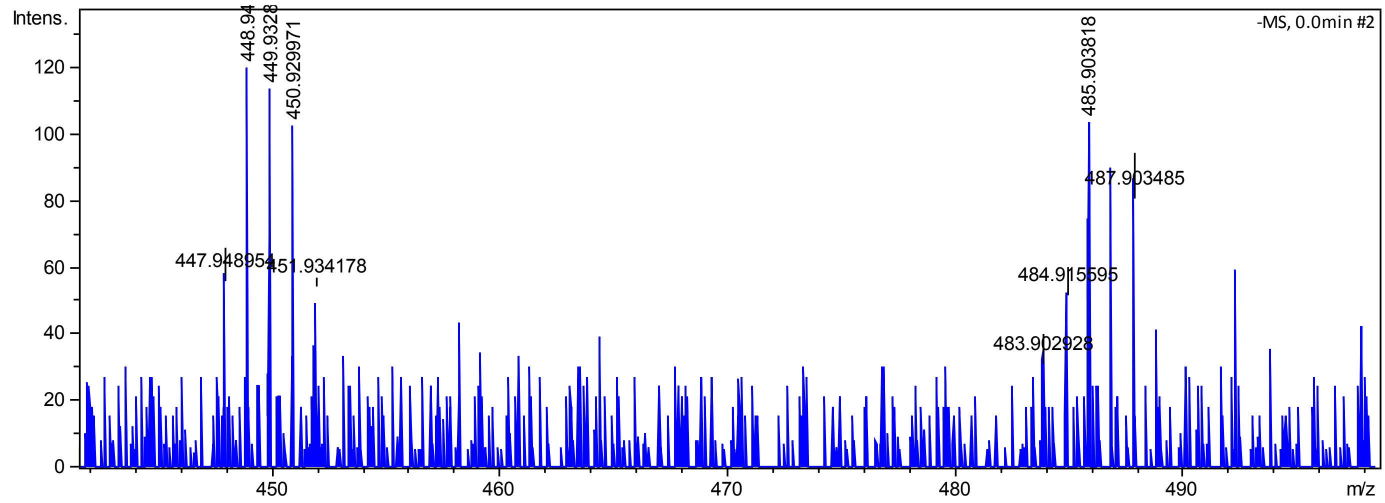

Fig.12 Experimental ESI-MS (negative ionization in MeOH) spectrum of the compound **8**

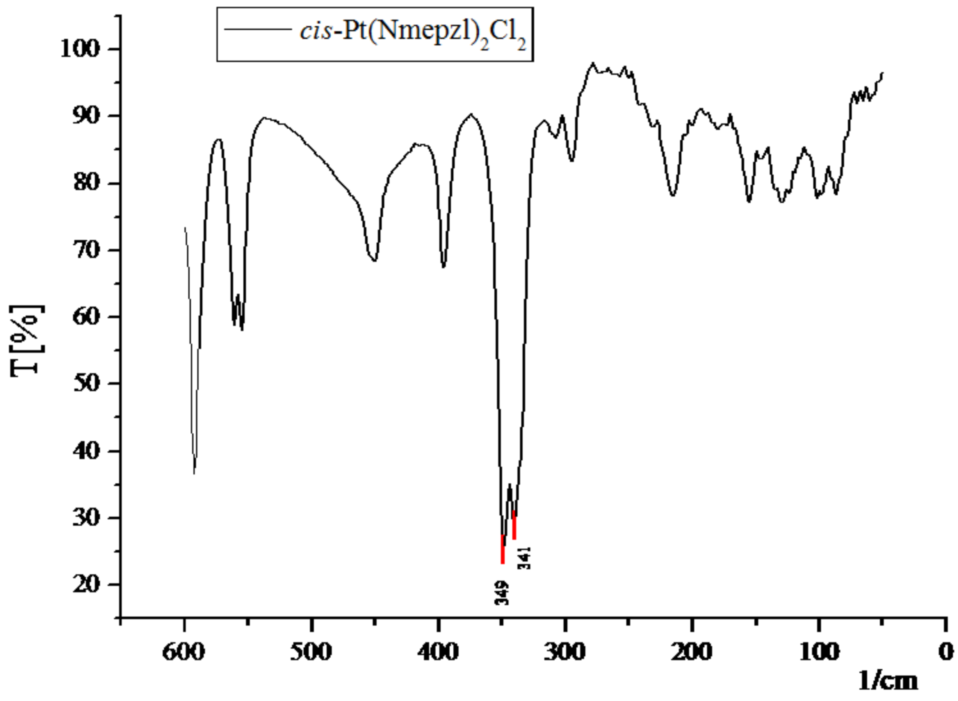

Fig.S13 Experimental far IR spectrum of the *cis* complex **1**

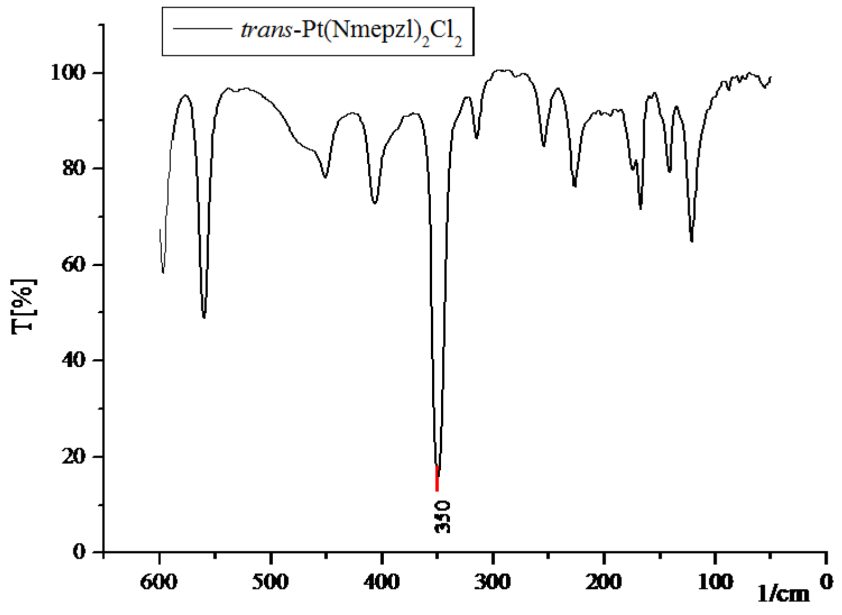

Fig.S14 Experimental far IR spectrum of the *trans* complex **2**

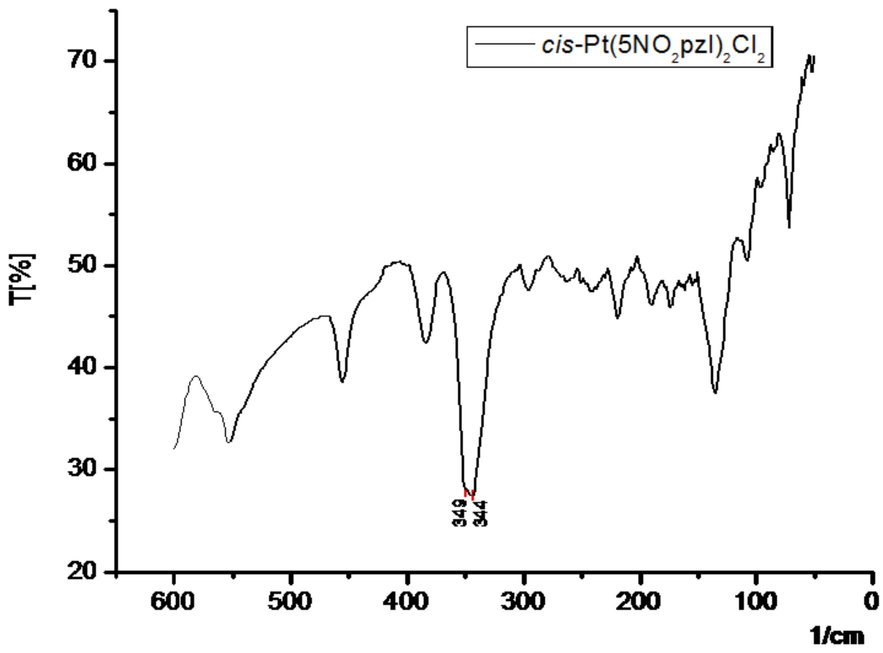

Fig.S15 Experimental far IR spectrum of the *cis* complex **3**

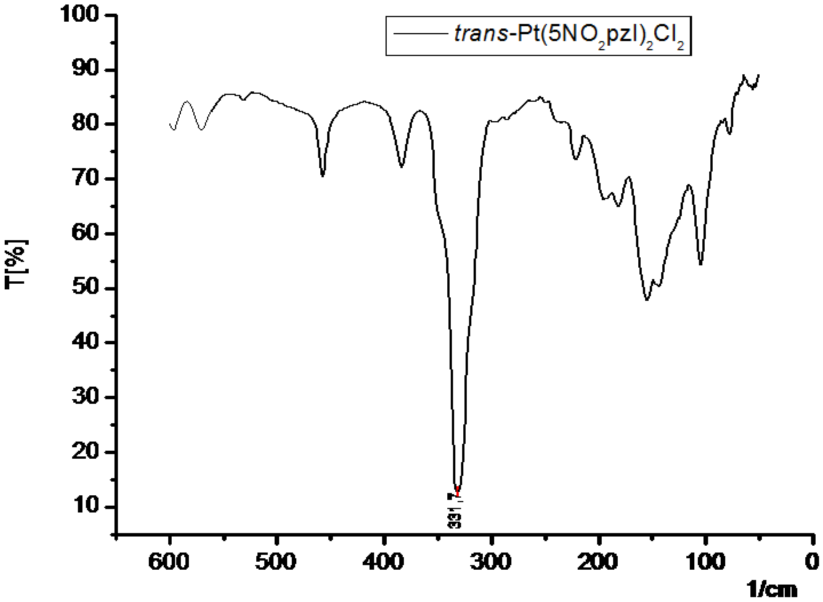

Fig.S16 Experimental far IR spectrum of the *trans* complex **4**

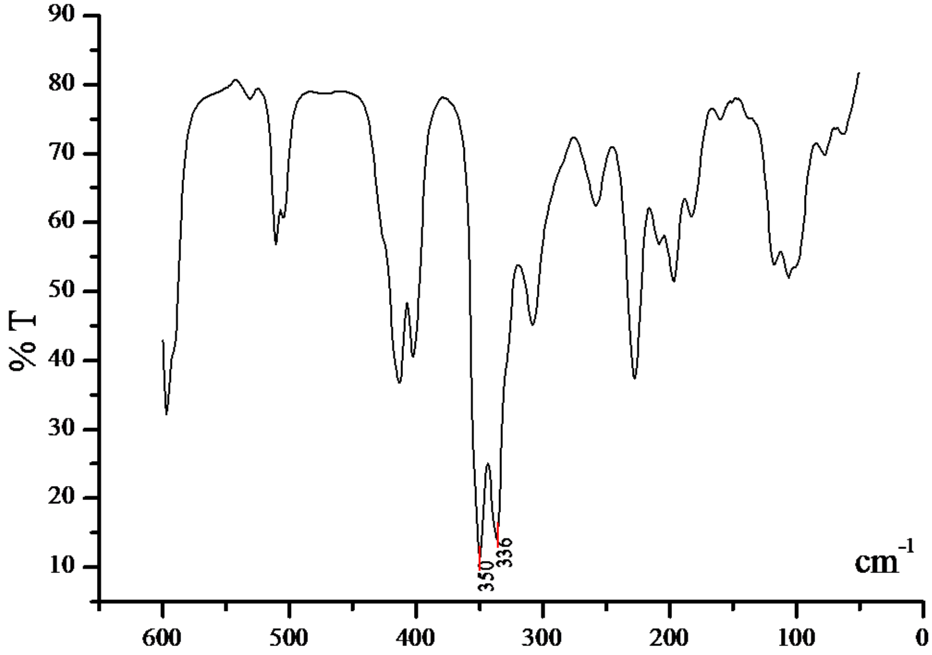

Fig.S17 Experimental far IR spectrum of the *cis* complex **5**

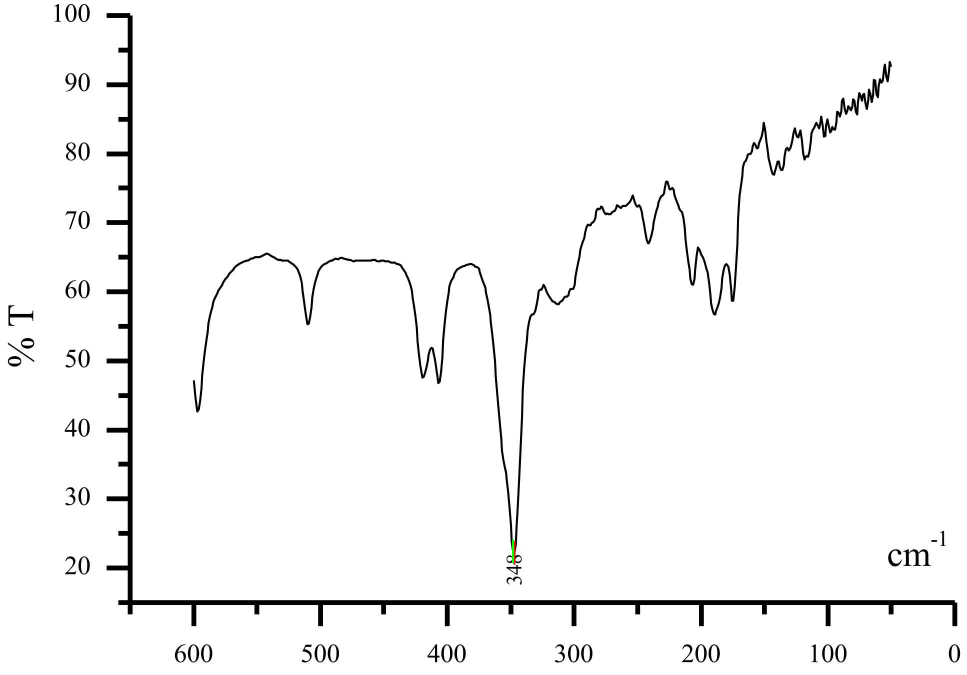

Fig.S18 Experimental far IR spectrum of the *trans* complex **6**

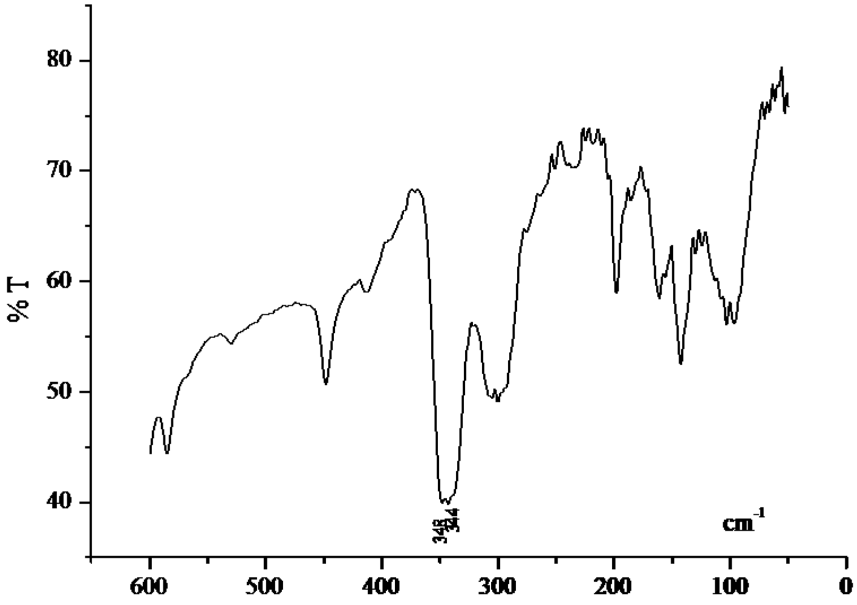

Fig.S19 Experimental far IR spectrum of the *cis* complex **7**

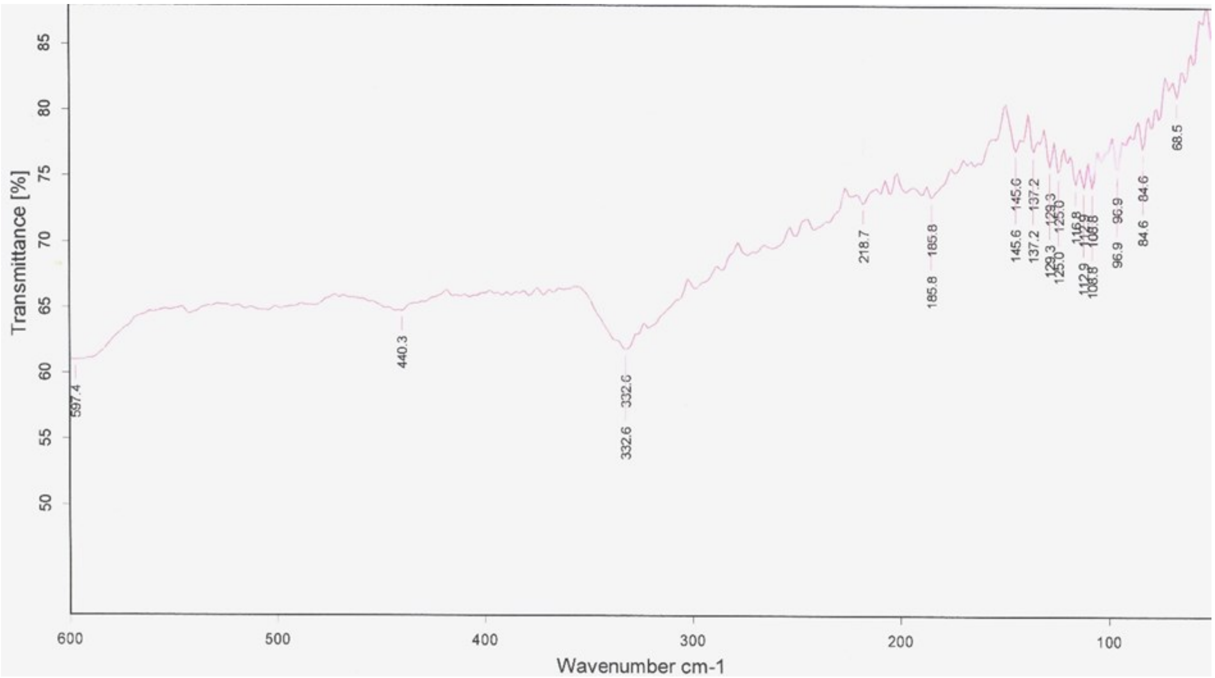

Fig.S20 Experimental far IR spectrum of the compound **8**

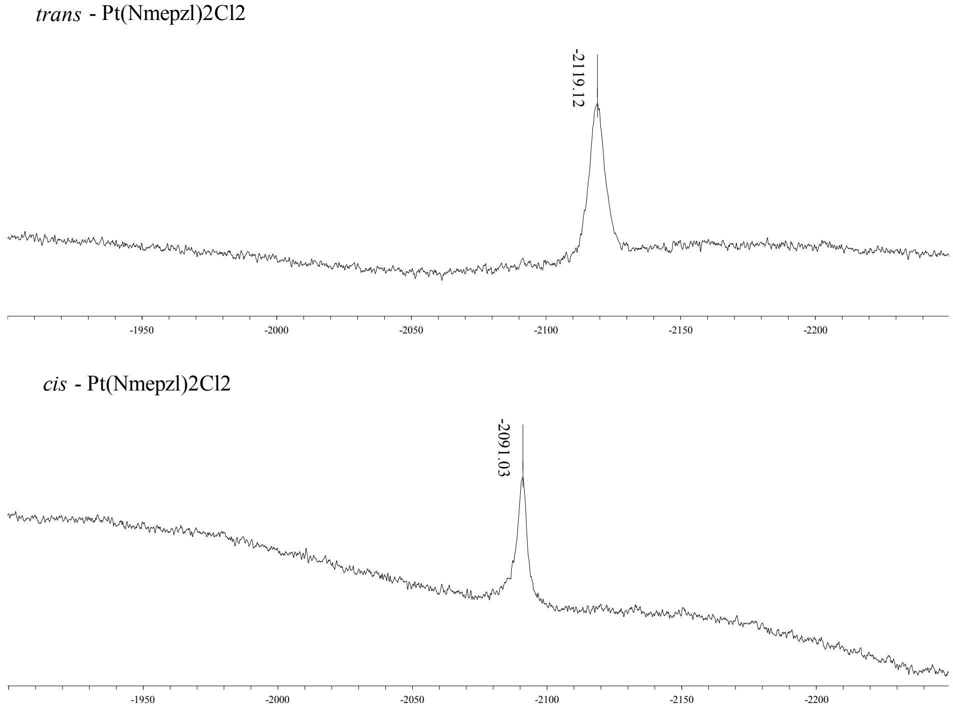

Fig. S21 ^{195}Pt NMR (acetone-d_6) spectra of *cis*- and *trans*-$Pt(Nmepzl)_2Cl_2$ (**1** and **2** respectively)

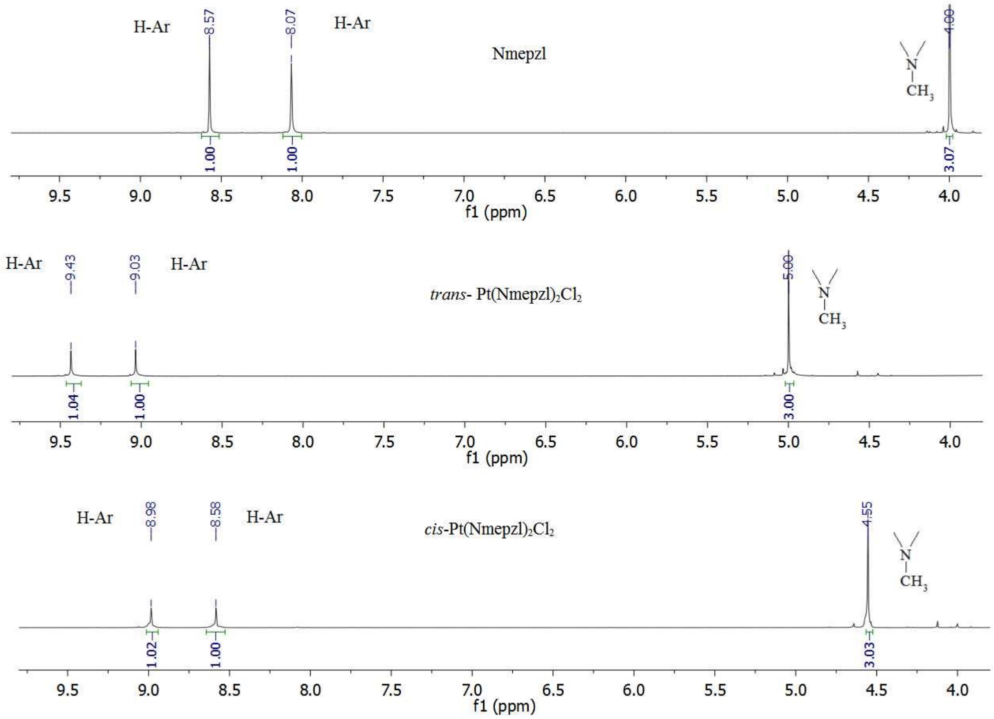

Fig. S22 Experimental ^{1}H NMR (acetone-d_6) spectra of *cis*- and *trans*-$Pt(Nmepzl)_2Cl_2$ (compounds **1** i **2** respectively) in comparison to 1-methyl-4-nitropyrazole.

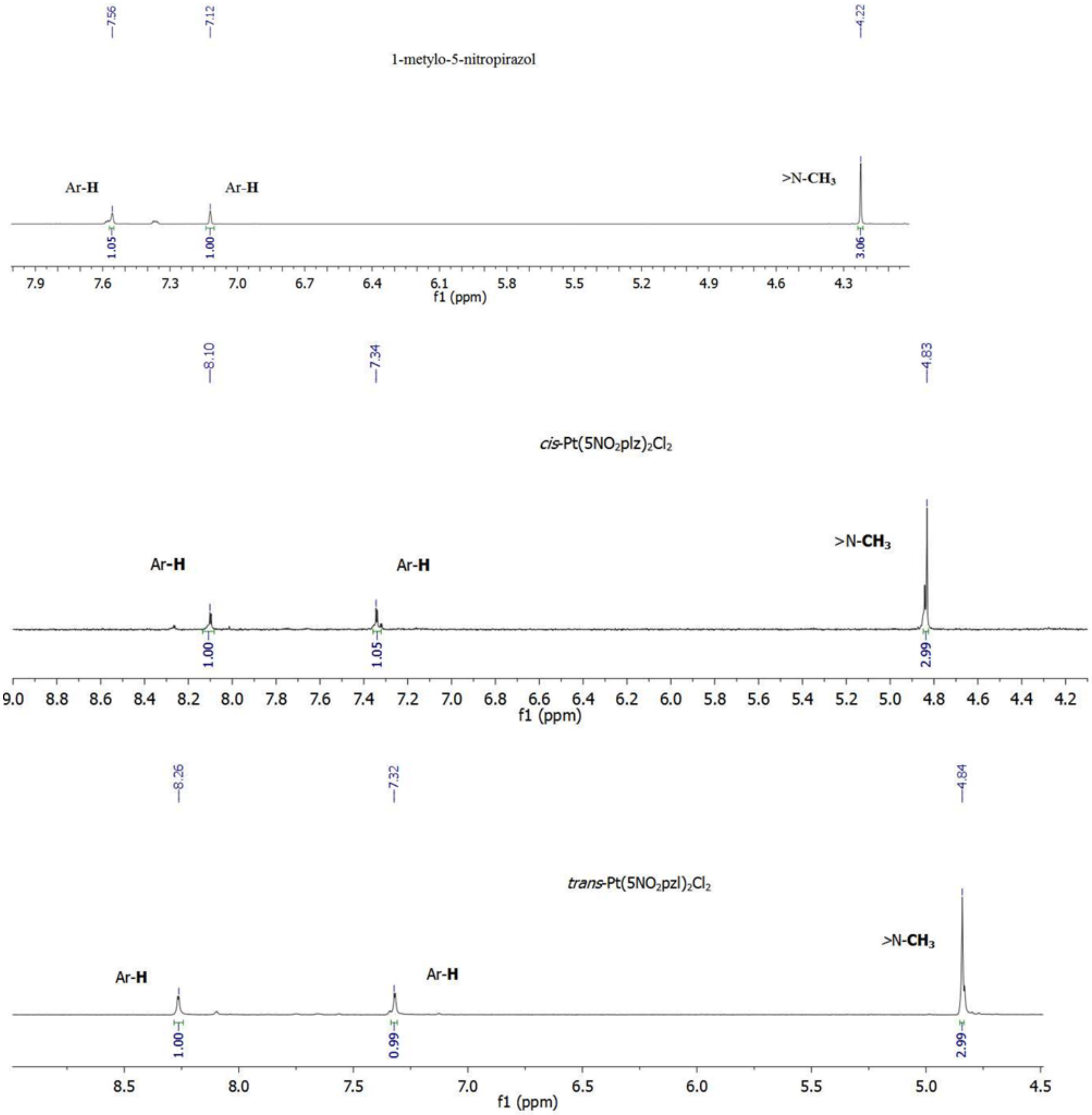

Fig. S23 Experimental ^{1}H NMR (acetone-d_6) spectra of *cis*- and *trans*-Pt(5NOpzl)$_2$Cl$_2$ (compounds **3** and **4** respectively) in comparison to 1-methyl-5-nitropyrazole.

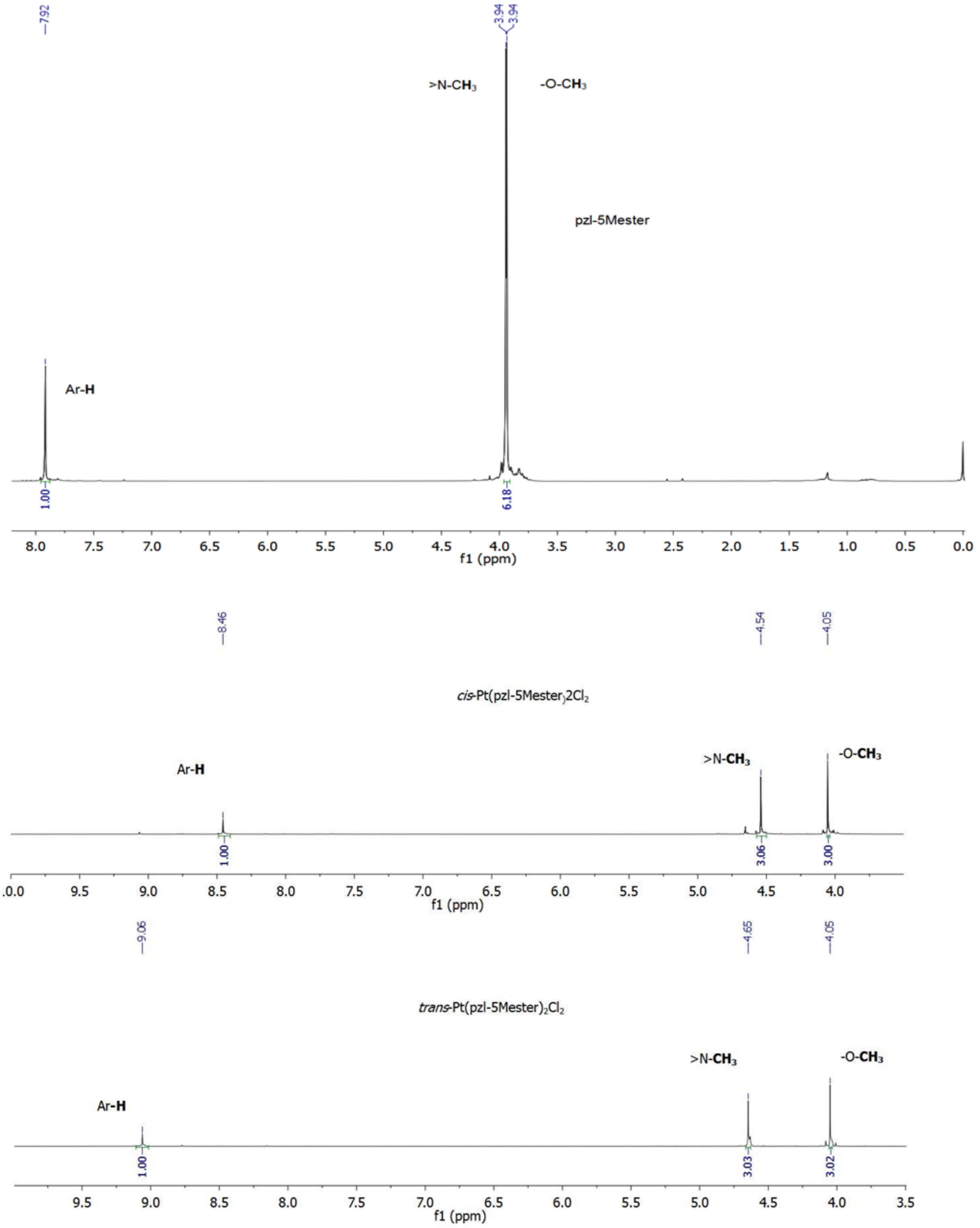

Fig. S24 Experimental ^{1}H NMR (acetone-d_6) spectra of *cis*- and *trans*-Pt(pzl-5-Mester)$_2$Cl$_2$ (compounds **5** and **6** respectively) in comparison to 1-methyl-4-nitropyrazole-5-carboxylic acid methyl ester.

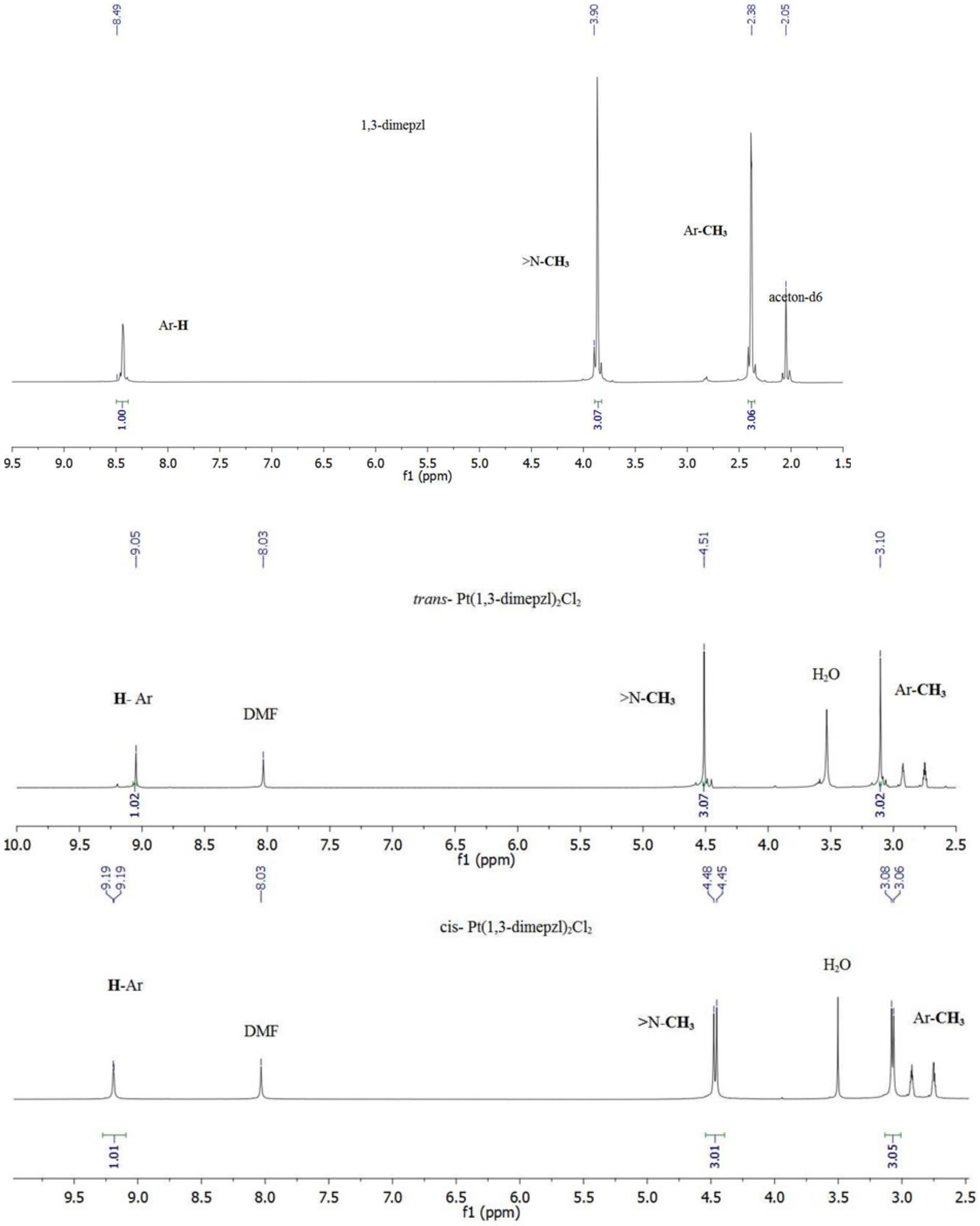

Fig. S25 Experimental ^{1}H NMR (DMF-d_7) spectra of *cis*- (1,3-dimepzl)$_2$Cl$_2$ (compound **7**) in comparison to its trans- congener and 1,3-dimethyl-5-nitropyrazole.

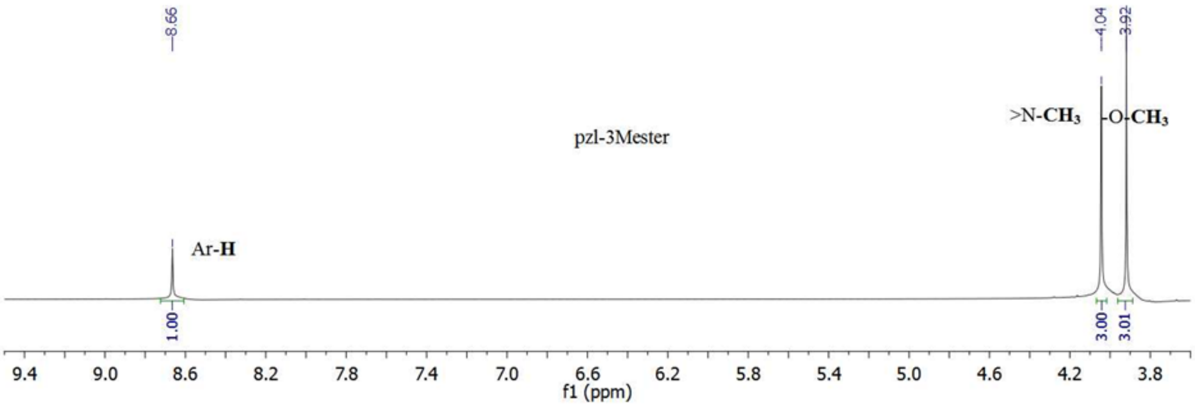

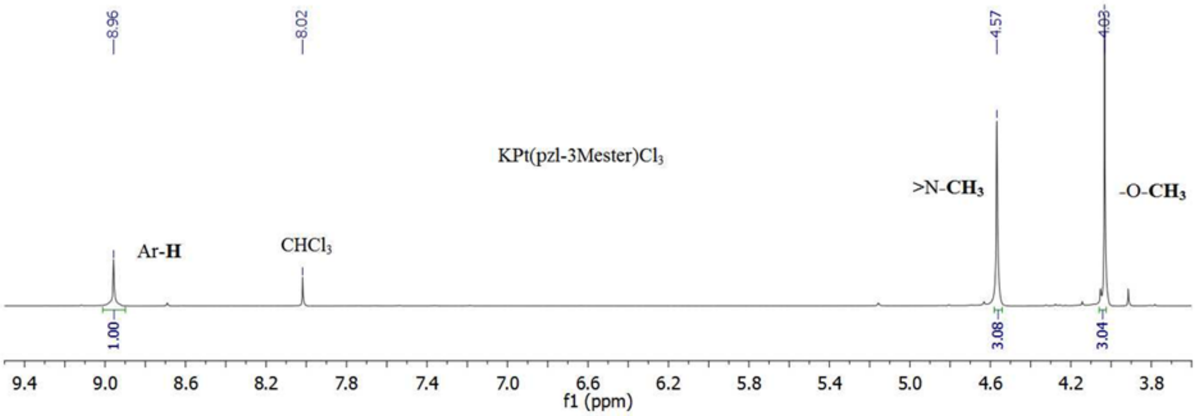

Fig. S26 Experimental ^{1}H NMR (acetone-d_6) spectra of KPt(pzl-3Mester)Cl_3 (compound **8**) and 1-methyl-4-nitro-3-carboxylic acid methyl ester.

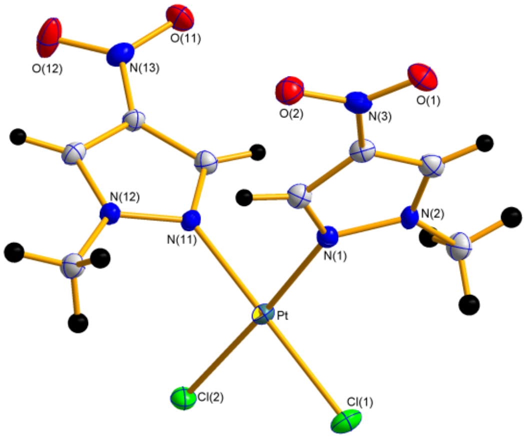

Fig. S27 Molecular crystallographic structure determined on the basis of X-ray diffraction (XRD) for cis-dichloridobis(1-methyl-4-nitropyrazole)platinum(II) **1**. Thermal ellipsoids are drawn at the 50% probability level.

Table 5. Selected X-ray Data for Compound **1**

Compound No	1
**Formula**	**$C_8H_{10}Cl_2N_6O_4Pt$**
Formula weight	520.20
Temperature [K]	100(2)
λ [Å]	0.71073
Crystal system	Monoclinic
Space group	C2/c(No.15)
a [Å]	25.032(3)
b [Å]	9.461(3)
c [Å]	15.187(5)
α [°]	
β [°]	126.94(3)
γ [°]	
V [$Å^3$]	2874.7(18)
Z, ρ_{calc} [g cm^{-3}]	8, 2.404
μ [mm^{-1}]	10.158
F(000)	1952
Crystal size [mm]	0.10x0.08x0.05
θ range[º]	3.25-36.89
rflns: total /unique	21034/6316
Abs. corr.	analytical
Min., max. transmission factors	0.789/810
Data/restraints/params	6316/0/192
GOF on F^2	0.901
R_1 [$I > 2\sigma(I)$]	0.0286
wR_2 (all data)	0.0453
Max., min. $\Delta\rho_{elect}$ [e $Å^3$]	1.150/-1.426

CCDC reference number for compound **1**: CCDC924455

For the crystal **1** data collection were carried out using a KM4-CCD diffractometer, ω scans, and graphite-monochromated Mo-Kα radiation generated from a diffraction X-ray tube operating at 50 kV and 25 mA. Data were corrected for Lorentz and polarization effects. Absorption corrections were performed for the intensity data (Tmin = 0.678 and Tmax = 0.789) with CrysAlisPro data collection and processing software (Oxford Diffraction, CrysAlis RED, CrysAlisCCD (Version 1.171.30); Oxford Diffraction Ltd., Abingdon, Oxfordshire, UK (2004)). The structure was solved by direct methods (SHELXS97) [1] and refined by the full-matrix least-squares method on all F2 data (SHELXL97) [2]. The atoms H atoms were included from the geometry of molecules and were not refined. Crystal data and details of data collection and refinement procedures are collected in **Table 5**. The reflection intensities were treated by the PLATON program (version 281019) with 'squeeze' procedure, because the position of a solvent molecule was not determined [3].

[1] SHELXS97, G. M. Sheldrick, SHELXS97, Program for the Solution of Crystal Structures; University of Göttingen, Germany (1997).
[2] SHELXL97, G. M. Sheldrick, SHELXL97, Program for the Refinement of Crystal Structures; University of Göttingen, Germany.
[3] Spek, A. L. (2009), Acta Cryst. D65, 148-159 *PLATON. A Multipurpose Crystallographic Tool*. Utrecht University, The Netherlands. (http://www.cryst.chem.uu.nl/platon.)
